# Cell type-specific epigenetic links to schizophrenia risk in the brain

**DOI:** 10.1186/s13059-019-1747-7

**Published:** 2019-07-09

**Authors:** Isabel Mendizabal, Stefano Berto, Noriyoshi Usui, Kazuya Toriumi, Paramita Chatterjee, Connor Douglas, Iksoo Huh, Hyeonsoo Jeong, Thomas Layman, Carol A. Tamminga, Todd M. Preuss, Genevieve Konopka, Soojin V. Yi

**Affiliations:** 1 School of Biological Sciences, Georgia Institute of Technology, Atlanta, GA 30332 USA; 2 Department of Neuroscience, UT Southwestern Medical Center, Dallas, TX 75390 USA; 3 Department of Psychiatry, UT Southwestern Medical Center, Dallas, TX 75390 USA; 4 Division of Neuropharmacology and Neurologic Diseases, Department of Pathology, Yerkes National Primate Research Center, Emory University School of Medicine, Emory University, Atlanta, GA 30329 USA; 5 Center for Medical Research and Education, Graduate School of Medicine, Osaka University, Suita, Osaka 565-0871 Japan; 6 Department of Neuroscience and Cell Biology, Graduate School of Medicine, Osaka University, Suita, Osaka 565-0871 Japan; 7 Schizophrenia Research Project, Department of Psychiatry and Behavioral Sciences, Tokyo Metropolitan Institute of Medical Science, Tokyo, 156-8506 Japan; 8 College of Nursing, The Research Institute of Nursing Science, Seoul National University, Seoul, 03080 South Korea

**Keywords:** Schizophrenia, Neurogenomics, Epigenetics, DNA methylation, Transcriptome, Brain cell type, Neuron, Oligodendrocyte

## Abstract

**Background:**

The importance of cell type-specific epigenetic variation of non-coding regions in neuropsychiatric disorders is increasingly appreciated, yet data from disease brains are conspicuously lacking. We generate cell type-specific whole-genome methylomes (*N* = 95) and transcriptomes (*N* = 89) from neurons and oligodendrocytes obtained from brain tissue of patients with schizophrenia and matched controls.

**Results:**

The methylomes of the two cell types are highly distinct, with the majority of differential DNA methylation occurring in non-coding regions. DNA methylation differences between cases and controls are subtle compared to cell type differences, yet robust against permuted data and validated in targeted deep-sequencing analyses. Differential DNA methylation between control and schizophrenia tends to occur in cell type differentially methylated sites, highlighting the significance of cell type-specific epigenetic dysregulation in a complex neuropsychiatric disorder.

**Conclusions:**

Our results provide novel and comprehensive methylome and transcriptome data from distinct cell populations within patient-derived brain tissues. This data clearly demonstrate that cell type epigenetic-differentiated sites are preferentially targeted by disease-associated epigenetic dysregulation. We further show reduced cell type epigenetic distinction in schizophrenia.

**Electronic supplementary material:**

The online version of this article (10.1186/s13059-019-1747-7) contains supplementary material, which is available to authorized users.

## Background

Schizophrenia is a lifelong neuropsychiatric psychotic disorder affecting 1% of the world’s population [1]. Genetic dissection of schizophrenia risk has revealed the polygenic nature of the disorder [2–4]. Many of the schizophrenia risk loci are located in the non-coding regions of the genome, suggesting gene regulation plays a role in disease pathology. Indeed, a large number of these risk loci are associated with alterations in the gene expression in schizophrenia [2, 5, 6]. These observations implicate epigenetic mechanisms as potential mediators of genetic risk in schizophrenia pathophysiology. Epigenetic mechanisms, such as DNA methylation, may have particular relevance for human brain development and neuropsychiatric diseases [7–9]. Previous studies found that changes in DNA methylation associated with schizophrenia are significantly enriched with DNA methylation changes from prenatal to postnatal life [7]. Moreover, genome-wide association studies (GWAS) of schizophrenia risk loci were over-represented in variants that affect DNA methylation in fetal brains [10].

Prior studies of the genetic and epigenetic risks for schizophrenia have some limitations, however, including the use of pre-defined microarrays, which traditionally focused on CpG islands and promoters, for methylation profiling. Unbiased, genome-wide analyses of DNA methylation are revealing that variation in DNA methylation outside of promoters and CpG islands define the critical epigenetic difference between diverse cell types [11, 12]. Additionally, previous genomic studies of schizophrenia have used brain tissue samples containing a heterogeneous mixture of cell types, although gene expression patterns vary considerably across cell types in the human brain [13–17]. To address these concerns, we carried out whole-genome methylome and transcriptome analyses using postmortem human brain tissue that underwent fluorescence-activated nuclei sorting (FANS) [18] into neuronal (NeuN^+^) and oligodendrocyte (OLIG2^+^) cell populations. Both neurons and myelin-forming oligodendrocytes have been implicated in schizophrenia pathophysiology [19, 20] and may be functionally dependent on one another for proper signaling in the brain [21]. Tissue was dissected from Brodmann area 46 (BA46) of the dorsolateral prefrontal cortex, a key brain region at risk in schizophrenia [1, 22]. We used whole-genome bisulfite sequencing (WGBS) to obtain an unbiased assessment of epigenetic modifications associated with schizophrenia and additionally carried out whole-genome sequencing (WGS) and RNA sequencing (RNA-seq) of the same samples to document transcriptomic consequences while accounting for the genetic background differences.

Integrating these data, we demonstrate extensive differential DNA methylation between neurons and oligodendrocytes. Comparisons to previous studies using bulk tissues indicate that they were generally biased toward neuronal populations. Our resource thus offers comprehensive and balanced analyses of molecular variation in control and disease brains, including novel information from a major yet relatively underexplored brain cell population (oligodendrocytes). This comprehensive and novel dataset allows us to demonstrate subtle yet robust DNA methylation differences between control and schizophrenia samples, which are highly enriched in sites that are epigenetically differentiated between the two cell types. Moreover, we show that schizophrenia-associated DNA methylation changes reduce the cell type methylation difference. Together, these data indicate that the integration of multiple levels of data in a cell type-specific manner can provide novel insights into complex genetic disorders such as schizophrenia.

## Results

### Divergent patterns of DNA methylation in human brain cell types

**Fig. 1:**
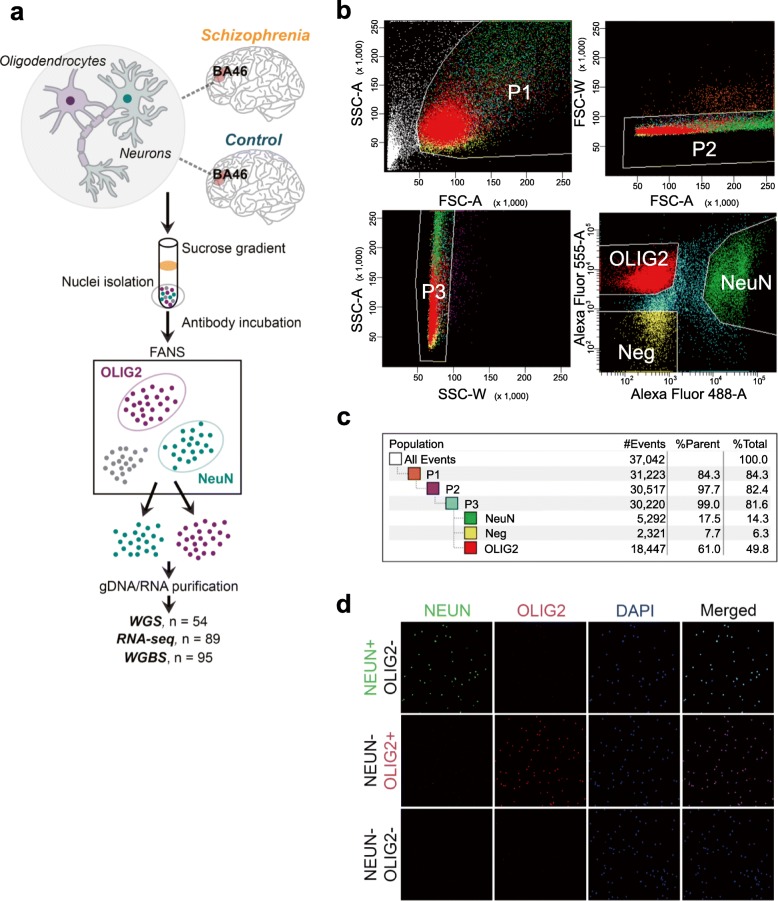
Experimental design and FANS workflow example. **a** Postmortem brain tissue from BA46 was matched between cases with schizophrenia and unaffected individuals. Tissue pieces were processed to isolate nuclei and incubated with antibodies directed toward NeuN or OLIG2. The nuclei were sorted using fluorescence-activated nuclei sorting (FANS) to obtain purified populations of cell types. The nuclei were processed to obtain genomic DNA (gDNA) and nuclear RNA from the same pools. Nucleic acids then underwent whole-genome sequencing (WGS), whole-genome bisulfite sequencing (WGBS), or RNA sequencing (RNA-seq). **b** NeuN-positive (NeuN^+^) nuclei represent neurons within the cerebral cortex as few human NeuN-negative (NeuN^−^) cells in the cortex are neurons [23, 24] (e.g., Cajal-Retzius neurons). OLIG2-positive (OLIG2^+^) nuclei represent oligodendrocytes and their precursors [25, 26]. Isolation of nuclei expressing either NeuN conjugated to Alexa 488 or OLIG2 conjugated to Alexa 555. The nuclei were first sorted for size and complexity, followed by gating to exclude doublets that indicate aggregates of nuclei and then further sorted to isolate nuclei based on fluorescence. “Neg” nuclei are those that are neither NeuN^+^ nor OLIG2^+^. **c** Example percentage nuclei at each selection step during FANS. Note that while in this example more nuclei were OLIG2^+^, in other samples, the proportions might be reversed. **d** Immunocytochemistry of nuclei post-sorting. The nuclei express either NeuN or OLIG2 or are negative for both after FANS. DAPI labels all nuclei

We performed FANS [18] using postmortem tissue dissected from BA46 of the dorsolateral prefrontal cortex using NeuN and OLIG2 antibodies (Fig. 1a; Additional file 1: Table S1; see the “Methods” section). Immunofluorescent labeling indicates that NeuN-positive nuclei and OLIG2-positive nuclei following FANS (hereinafter “NeuN^+^” or “OLIG2^+^”) represent neurons within the cerebral cortex and oligodendrocytes and their precursors, respectively (Fig. 1b–d). We analyzed genomic DNA (gDNA) and total RNA from the same nuclei preparations in NeuN^+^ or OLIG2^+^ by WGBS and RNA-seq. We additionally carried out WGS of the brain samples to explicitly account for the effect of genetic background differences.

**Fig. 2:**
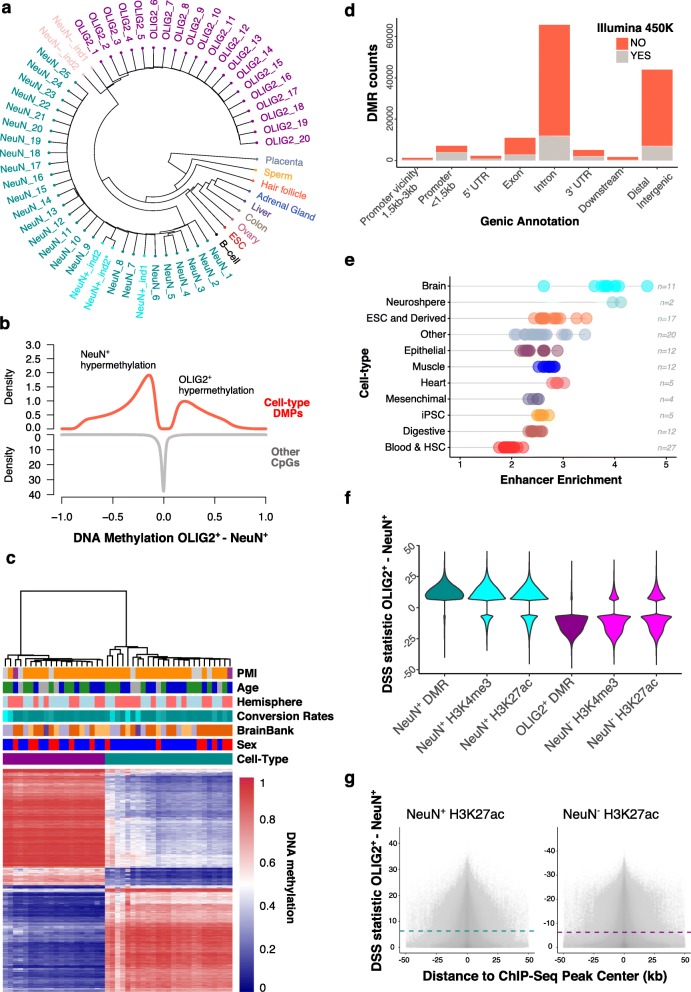
Divergent patterns of DNA methylation in NeuN^+^ and OLIG2^+^ cell types in the human brain. **a** Clustering analysis based on whole-genome CpG methylation values completely discriminated between NeuN^+^ (*N* = 25) and OLIG2^+^ (*N* = 20) methylomes. Additional NeuN^+^ (colored in turquoise) and those labeled as NeuN^−^ (pink) are from [27]. **b** Density plots showing the distribution of fractional methylation differences between OLIG2^+^ and NeuN^+^ at differentially methylated positions (DMPs) and other CpGs (non-DMPs). We observed a significant excess of NeuN^+^-hypermethylated DMPs compared to OLIG2^+^ (binomial test with expected probability = 0.5, *P* < 10^−15^). **c** Heatmap of the top 1000 most significant DMRs between OLIG2^+^ and NeuN^+^. Fractional methylation values per individual (column) and DMR (row) show substantial differences in DNA methylation and clear cell type clustering. **d** Genic annotation of DMRs and coverage with Illumina 450K Methylation Arrays. Counts of different genic positions of DMRs are shown. DMRs containing at least one CpG covered by a probe in the array are indicated. Only 20.8% of the DMRs contain one or more CpG targeted by Illumina 450K Array probes. **e** DMRs are enriched for brain enhancers. Enrichment of enhancer states at DMRs compared to the 100 matched control DMR sets from 127 tissues [28]. Random sets are regions with similar characteristics as, including the total number of regions, length, chromosome, and CG content. **f** Correspondence between cell type-specific methylation sites in NeuN^+^ and OLIG2^+^ with NeuN^+^ and NeuN^−^ ChIP-seq datasets [9]. Neuron-specific ChIP-seq peaks show an excess of sites with NeuN^+^-specific hypomethylated sites (positive DSS statistic, see the “Methods” section) whereas non-neuron peaks showed significant enrichment for sites specifically hypomethylated in OLIG2^+^ (negative DSS statistic). **g** Distribution of cell type-specific methylation differences at CpGs within H3K27ac ChIP-seq peaks in NeuN^+^ and NeuN^−^ nuclei. Positive values of DSS statistic indicate hypomethylation in NeuN^+^ compared to OLIG2^+^, whereas negative values indicate hypermethylation (see the “Methods” section). Dashed lines indicate the significance level for DSS analyses

Whole-genome DNA methylation maps of NeuN^+^ (*N* = 25) and OLIG2^+^ (*N* = 20) from control individuals (Additional file 1: Table S1) show a clear separation of the two populations (Fig. 2a). Previously published whole-genome methylation maps of neurons [27] co-segregate with NeuN^+^. On the other hand, previously generated NeuN^−^ methylomes [27] cluster as outliers of OLIG2^+^ samples, potentially due to the inclusion of other cell types compared to our cell-sorted samples. We identified differentially methylated CpGs between cell types, which we refer to as “differentially methylated positions (DMPs),” using a statistical method that allows us to explicitly take into account the effect of covariates (Additional file 1: Table S2; see the “Methods” section), while handling variance across biological replicates as well as the beta-binomial nature of read count distribution from WGBS [29]. Despite the large number of CpGs (~ 25 million out of the total 26 million CpGs in the human genome have been analyzed), we identify numerous DMPs between NeuN^+^ and OLIG2^+^ after correcting for multiple testing. At a conservative Bonferroni *P* < 0.05, over 4 million CpGs are differentially methylated between these two cell types, revealing highly distinct cell type difference in gDNA methylation (Fig. 2a, b). On average, DMPs between NeuN^+^ and OLIG2^+^ exhibit a 32.6% methylation difference. NeuN^+^ tends to be more hypermethylated than OLIG2^+^ (Fig. 2b; 64% of DMPs, binomial test, *P* < 10^−16^). This observation is consistent with NeuN^+^ being more hypermethylated than non-neuronal populations [27].

As expected from regional correlation of DNA methylation between adjacent sites [30–32], many DMPs occur near each other, allowing us to identify “differentially methylated regions” or “DMRs” (defined as ≥ 5 significant DMPs in a 50-bp region) spanning 103 MB in the human genome, exhibiting mean methylation difference of 38.3% between cell types (Fig. 2c, Additional file 2: Table S3). Many DMRs reside in introns and distal intergenic regions (Fig. 2d), which are traditionally viewed as “non-coding.” Chromatin state maps based on six chromatin marks [28] indicate that many DMRs, especially those located in introns and distal intergenic regions, exhibit enhancer chromatin marks, in particular, brain enhancers (OR between 2.6- and 4.6-fold, *P* < 0.01, Fig. 2e, Additional file 1: Table S4). In fact, over 60% of all DMRs show enhancer-like chromatin features in the brain (Additional file 3: Figure S1). These results highlight the regulatory significance of non-coding regions of the genome. Notably, currently available arrays such as the Illumina 450K do poorly in terms of targeting putative epigenetic regulatory loci (Fig. 2d).

NeuN^+^-specific hypo-methylated regions are significantly enriched in recently identified NeuN^+^-specific H3K4me3 and H3K27ac peaks [9] (Fig. 2f; Fisher’s exact test OR = 7.8, *P* < 10^−15^). H3K4me3 and H3K27ac peaks in the NeuN^−^ populations also show significant enrichment of OLIG2^+^-specific hypo-methylation, although the degree of enrichment is less strong than NeuN^+^ correspondence (Fisher’s exact test OR = 4.8, *P* < 10^−15^), again potentially due to the inclusion of other types of cells. WGBS data is complementary to ChIP-seq data in terms of resolution and coverage. While ChIP-seq provides resolution in the scale of several thousand base pairs (for example, peak sizes in previous study [9] are on average several kilobases and extend up to several hundred kilobases), WGBS data offers base pair resolution. Even though DMPs are generally concentrated around the center of ChIP-seq peaks, some peaks show more diffuse patterns, indicating that incorporating DMP information could offer fine-scale resolution of histone modification in individual genomic regions (Fig. 2g, Additional file 3: Figure S2).

We further examined DNA methylation of cytosines that are not in the CpG context, as nucleotide resolution whole-genome DNA methylation maps have begun to reveal the potential importance of non-CG methylation (CH methylation, where H = A, C, or T) particularly in neuronal function [27]. We observed that low levels of CH methylation were present in NeuN^+^ but nearly absent in OLIG2^+^ (Additional file 3: Figure S3), consistent with previous reports [27]. CH methylation is primarily associated with CA nucleotides (69.4%), followed by CT (26%) and CC (4.6%) (Additional file 3: Figure S3). In addition, gene body mCH values negatively correlate with gene expression in NeuN^+^ (Spearman’s rho − 0.16, *P* < 10^−10^; Additional file 3: Figure S3). Therefore, CH patterns at gene bodies provide an additional layer of gene expression regulation that is specific to neurons while absent in oligodendrocytes in the human brain.

### Strong association between cell type-specific DNA methylation and expression

**Fig. 3:**
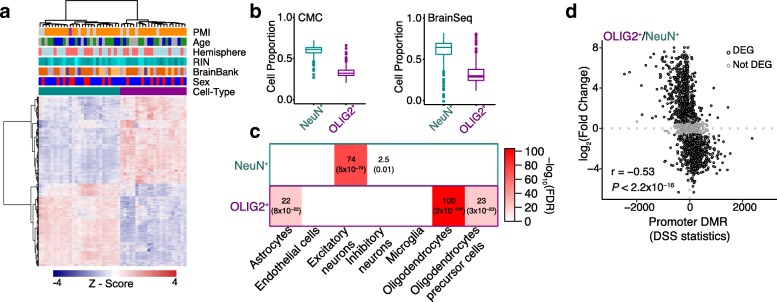
Gene expression signatures in NeuN^+^ and OLIG2^+^ nuclei. **a** Heatmap of cell type DEGs with covariates indicated. **b** Cell deconvolution of bulk RNA-seq data from the CommonMind Consortium and BrainSeq compared with NeuN^+^ and OLIG2^+^ (control samples). Y-axes show the weighed proportion of cells that explain the expression of bulk RNA-seq. **c** Gene set enrichment for cell type markers from single-nuclei RNA-seq. Enrichment analyses were performed using Fisher’s exact test. Odds ratios and FDRs (within parentheses) are shown. **d** Correspondence between the expression change and methylation change in cell types. The *X*-axis represents differential DNA methylation statistics for genes harboring DMRs in promoters. The *Y*-axis indicates the log_2_(fold change) of expression between the two cell types. The negative correlation supports the well-established impact of promoter hypomethylation on the upregulation of gene expression

We next performed RNA-seq using RNAs extracted from the nuclei-sorted populations (see the “Methods” section). NeuN^+^ and OLIG2^+^ transcriptomes form distinctive clusters (Fig. 3a). Transcriptomic data from cell-sorted populations clustered closer to bulk RNA-seq data from the cortical regions but were distinct from those from the cerebellum and whole blood (Additional file 3: Figure S4). We further show that previously generated bulk RNA-seq data [5, 6] have higher proportion of NeuN^+^ compared with OLIG2^+^ (Fig. 3b), indicating that these previously generated bulk RNA-seq data are biased toward neurons. The higher neuronal proportion in bulk RNA-seq is highlighted also using an independent single-nuclei data (Additional file 3: Figure S5).

We show that 55% of genes show significant change in expression between NeuN^+^ and OLIG2^+^ (|log_2_(fold change)| > 0.5 and Bonferroni correction < 0.05; Additional file 1: Table S5). NeuN^+^- and OLIG2^+^-specific genes (defined as significantly upregulated in NeuN^+^ compared to OLIG2^+^ and vice versa) are enriched for known markers of specific cell types of the brain. Specifically, NeuN^+^-specific genes are enriched for excitatory and inhibitory neurons, whereas OLIG2^+^-specific genes show strong enrichment for oligodendrocytes and lower enrichment for oligodendrocyte precursor cells and astrocytes (Fig. 3c). Divergent DNA methylation between cell types can explain a large amount of gene expression variation between cell types (Fig. 3d, Spearman’s rho = − 0.53, *P* < 10^−15^). Significant correlation extends beyond the promoter regions (Additional file 3: Figure S6),

### Differential DNA methylation associated with schizophrenia

We next analyzed whole-genome methylation maps from brain tissue from patients with schizophrenia (28 NeuN^+^ and 22 OLIG2^+^) and contrasted these data with data from matched controls (25 NeuN^+^ and 20 OLIG2^+^; see the “Methods” section) described in the previous section. Compared to the robust signal of cell type difference, DNA methylation changes associated with schizophrenia are subtler. At a moderately stringent FDR < 0.2, we identify 261 individual CpGs (60 in NeuN^+^ and 201 in OLIG2^+^) that are differentially methylated between control and schizophrenia. Applying additional filtering for high-coverage sites (20× in at least 80% of samples per disease-control group), we identify a total of 97 CpGs (14 NeuN^+^ and 83 OLIG2^+^ specific) at FDR < 0.2 (Additional file 1: Tables S6–S7). Results of differential DNA methylation analyses in the rest of the paper all refer to those from the filtered dataset, and differentially methylated sites between case and control are referred to as “szDMPs.” The average methylation difference between schizophrenia and control at FDR < 0.2 szDMPs is ~ 6% (Additional file 1: Tables S6–S7), which is within the range of case/control differences our sample sizes are empowered to detect according to our power analyses (see the “Methods” section; Additional file 3: Figure S7). The majority of the szDMPs (FDR < 0.2) are intronic (50.5%) and distal intergenic CpGs (45.4%), whereas only two of them are located within 3 kb from the transcriptional start sites (Additional file 1: Tables S6–S7). Interestingly, two szDMPs (FDR < 0.2) in OLIG2^+^ are located within the regions reported to be associated with schizophrenia by GWAS [4] including a CpG located in the intron of *NT5C2* gene, involved in purine metabolism.

**Fig. 4:**
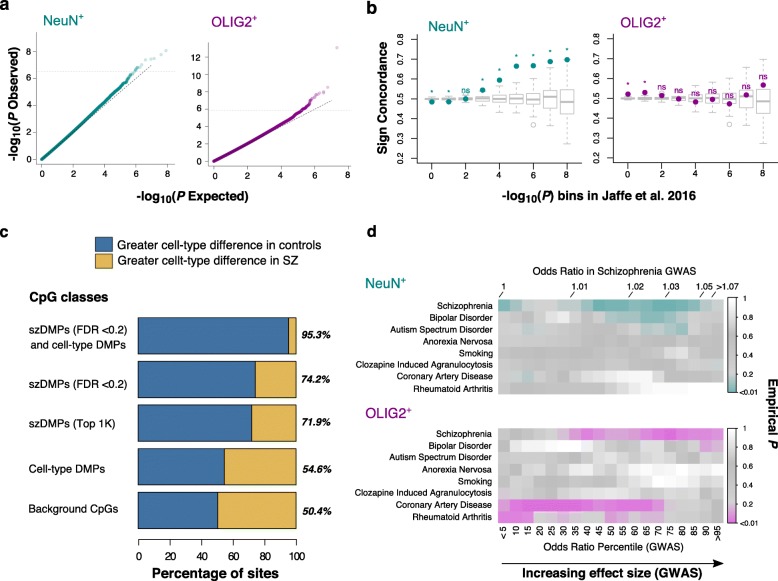
Cell type DNA methylation patterns associated with schizophrenia. **a** DMPs associated with schizophrenia. Quantile-quantile plots of genome-wide *P* values for differential methylation between schizophrenia and control based on NeuN^+^ (left) and OLIG2^+^ (right) WGBS data. **b** Concordance between WGBS data and microarray-based data. *Y*-axis shows the ratio of sites showing the concordant direction in schizophrenia vs. control in our study at each *P* value bin compared with the Jaffe et al. study [7] (*X*-axis). Concordance was tested using a binomial test (stars indicate *P* < 0.05). Boxplots correspond to the directional concordance in 100 sets of association results after case-control label permutations. NeuN^+^ (left) and OLIG2^+^ (right). **c** szDMPs show altered cell type differences. Barplot shows the percentage of sites with larger cell type differences in controls than in schizophrenia and vice versa at different CpG classes. Absolute OLIG2^+^ vs. NeuN^+^ methylation differences are larger in controls than cases in szDMPs compared to cell type DMPs and non-DMP or background CpGs. szDMPs were detected as differentially methylated between cases and controls at FDR < 0.2 in NeuN^+^ (14 sites) and OLIG2^+^ samples (83 sites). Top 1000 szDMPs were selected as the top 1000 loci according to best *P* values in each cell type (*N* = 2000). Cell type DMPs were detected by comparing OLIG2^+^ vs. NeuN^+^ methylomes at Bonferroni *P* < 0.05. Background CpGs were sampled from CpGs showing non-significant *P* values for both case-control and OLIG2^+^ vs. NeuN^+^ comparisons. Stars represent *P* values for binomial tests with all comparisons showing *P* < 10^−7^. **d** Top 1000 szDMPs are enriched for SZ GWAS signals. szDMPs identified in our methylation study in both cell types consistently co-localize with genetic variants with moderate to large effect sizes for schizophrenia risk than expected. The table shows the empirical *P* values of szDMPs at each odds ratio (OR) percentile of different traits from genome-wide association studies (GWAS). The actual ORs corresponding to the schizophrenia percentiles are indicated at the top. Specifically, for each szDMP, we identified all SNPs reported by the GWAS study within a 1-kb window and counted the number of SNPs at different quantiles of odds ratio (OR). We used quantiles of OR so that we can compare the different diseases and traits among them. We repeated this step using the same number of random non-szDMPs 100 times. To obtain empirical *P* values, we calculated the number of times non-szDMP sets showed more SNPs in each OR quantile than szDMPs. SNPs with moderate-to-high OR in schizophrenia GWAS consistently showed low empirical *P* values for both cell type DMPs, implying that SNPs with large effect sizes in GWAS studies are closer to szDMPs than expected. Interestingly, this pattern was not observed for other traits, implying the co-localization is exclusive to the disease

In addition to the power analysis (see the “Methods” section, Additional file 3: Figure S7), we assessed the robustness of the results as well as the effects of covariates or potential hidden structures in the data by permutation analysis, by randomly assigning case/control labels 100 times per cell type. The observed DNA methylation difference between control and schizophrenia samples is significantly greater than those observed in the permuted samples (Additional file 3: Figure S8). Even though our statistical cutoff is moderate, considering that we are correcting for an extremely large number of (~ 25 million) independent tests, the results from permutation analyses provide support that these sites represent schizophrenia-associated signals of differential DNA methylation. Indeed, quantile-quantile plots suggest that our data exhibit a modest but significant excess of good *P* values (Fig. 4a).

We also performed targeted experiments of 66 CpGs (16 szDMPs at FDR < 0.2 andNucleic Acids ResNucleic Acids Re 50 adjacent sites) through deep coverage sequencing using 24 samples from the discovery cohort as well as an additional 20 new independent samples. This validation analysis achieved an average read depth coverage of > 14,500×. Technical replicates are highly correlated with the fractional methylation values obtained from the WGBS (Spearman’s rho = 0.96, *P* < 10^−15^, Additional file 3: Figure S9), indicating the reliability of the fractional methylation estimates obtained in the discovery WGBS data. In addition, the WGBS data and validation data are highly consistent for case-control comparisons in both sign direction and correlation in effect size (Spearman’s rho = 0.87, *P* < 10^−16^ and 81.25% sign concordance, Additional file 3: Figure S10). These results support the validity of szDMPs discovered in our study.

There is no direct overlap between these DMPs (FDR < 0.2) and those previously identified from a microarray study [7]. However, despite the lack of direct overlap, the direction of methylation change between control and schizophrenia between the two studies is largely consistent in the NeuN^+^, especially with increasing significance (decreasing *P* values) (Fig. 4b). This pattern is highly significant compared to the permuted data (Fig. 4b). In comparison, the OLIG2^+^ dataset does not exhibit such a pattern (Fig. 4b), potentially because the bulk tissue samples consisted largely of neurons. Deconvolution analyses of transcriptomes using our cell-sorted population support this idea (Fig. 3b).

### Enrichment of szDMPs in cell type distinct sites imply cell type dysregulation

Remarkably, szDMPs (FDR < 0.2) are highly enriched in cell type-specific DMPs (OR = 4.1, *P* < 10^−10^, Fisher’s exact test). This enrichment persists when we examine a larger number of sites (Additional file 3: Figure S11), indicating that the enrichment is not due to the small number of szDMPs. Moreover, szDMPs (FDR < 0.2) show distinct directionality in the distinct brain cell types. Specifically, NeuN^+^ szDMPs (FRD < 0.2) show an excess of hypomethylation in schizophrenia samples compared to the control samples (93%, 13 out of 14, *P* = 0.0018 by binomial test, Additional file 3: Figure S8). An opposite pattern is observed for OLIG2^+^ szDMPs (FDR < 0.2), where schizophrenia samples are mostly hypermethylated compared to the control samples (75.9%, 63 out of 83, *P* = 2.4 × 10^−6^ by a binomial test). In contrast, this bias is not observed in the permuted data (NeuN^+^ empirical *P* = 0.07 and OLIG2^+^ empirical *P* = 0.02, Additional file 3: Figure S8). Considering that NeuN^+^ tend to be more hypermethylated compared to OLIG2^+^ (Fig. 2b), we investigated whether disease patterns in schizophrenia contribute to reduced cell type difference in DNA methylation. Indeed, szDMPs consistently show decreased cell type methylation difference compared to the control samples (Fig. 4c). In other words, schizophrenia-associated modification of DNA methylation effectively diminishes cell type distinctive epigenetic profiles in our data.

These results also suggest that sites that did not pass the FDR cutoff but have been detected in the differential methylation analyses may harbor meaningful candidates for future studies. Indeed, our power study supports this idea (see the “Methods” section, Additional file 3: Figure S7). Consequently, we further analyzed sites that are ranked top 1000 in the differential DNA methylation analysis between the brains of control vs. those from patients with schizophrenia (referred to as “top 1000” DMPs). We find that genes harboring the top 1000 szDMPs show enrichment for brain-related functions and diseases, as well as transcription factors, particularly those involved in chromatin remodeling (Additional file 3: Figure S12). Given that the majority of the schizophrenia heritability is found below the significance thresholds of GWAS [4], we explored the association patterns at genome-wide SNPs. Top 1000 szDMPs tend to co-localize with genetic variants associated with schizophrenia but not with other mental or non-mental traits, mostly with genetic variants below the strict GWAS significance threshold but with moderate-to-high effect sizes (Fig. 4d). This result supports the role of brain DNA methylation in the genetic etiology of schizophrenia.

### Cell type expression differences associated with schizophrenia

**Fig. 5:**
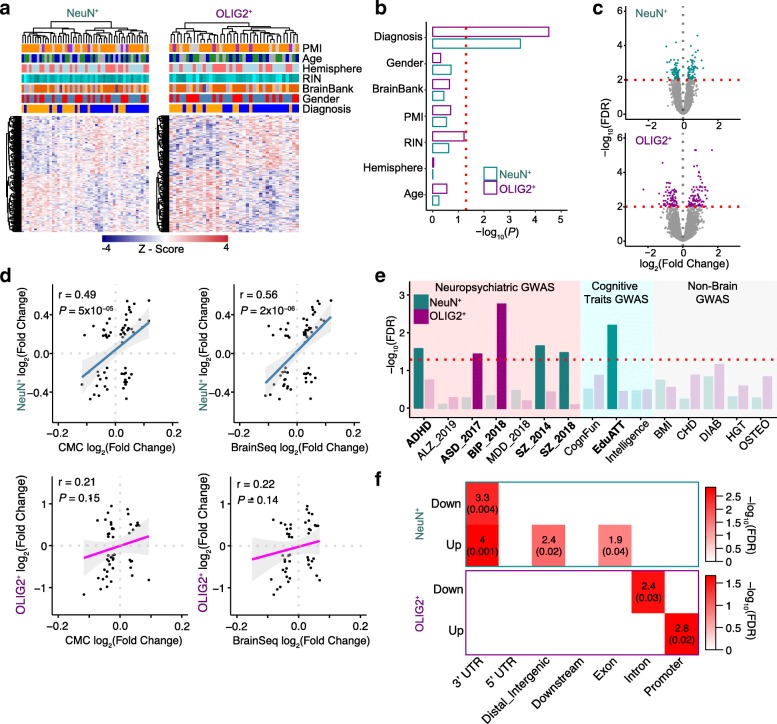
Gene expression associated with schizophrenia in NeuN^+^ and OLIG2^+^. **a** Heatmap of szDEGs for each cell type with covariates indicated. **b** The first principal component of the DEGs was associated with diagnosis. Red dotted line corresponds to *P* = 0.05. **c** Volcano plot showing szDEGs. *X*-axis indicates the log_2_(fold change), and *Y*-axis indicates log_10_(FDR). szDEGs (FDR < 0.01) are colored. **d** Comparisons of differentially expressed genes in schizophrenia from the current study with the BrainSeq and CMC data. We used genes that are classified as differentially expressed in all three datasets (each dot represents a gene, 63 genes are common to NeuN^+^, CMC, and BrainSeq, and 49 to OLIG2^+^, CMC, and BrainSeq). The *X*-axes represent the fold change between control and schizophrenia in CMC or BrainSeq datasets, and the *Y*-axes represent the log_2_(fold change) between control and schizophrenia in the current datasets, for either NeuN^+^-specific or OLIG2^+^-specific genes. Regression line and confidence interval are shown for each comparison. **e** Barplot highlighting the enrichment for trait-associated genetic variants. Bars correspond to NeuN^+^ (cyan) and OLIG2^+^ (magenta) szDEGs. Red dashed line corresponds to the FDR threshold of 0.05. *X*-axis shows the acronyms for the GWAS data utilized for this analysis (*ADHD*, attention deficit hyperactivity disorder; *ASD*, autism spectrum disorders; *BIP*, bipolar disorder; *ALZ*, Alzheimer’s disease; *MDD*, major depressive disorder; *SZ*, schizophrenia; *CognFun*, cognitive function; *EduAtt*, educational attainment; *Intelligence*, intelligence; *BMI*, body mass index; *CAD*, coronary artery disease; *DIAB*, diabetes; *HGT*, height; *OSTEO*, osteoporosis). **f** Enrichment map for szDEGs (up-/downregulated) and the top 1000 szDMPs (*X*-axis shows genic annotation). Enrichment analyses were performed using Fisher’s exact test. Reported odds ratios and FDRs within parentheses for NeuN^+^ (top) and OLIG2^+^ (bottom)

Compared to subtle DNA methylation differences, gene expression shows good separation between schizophrenia and control (Fig. 5a), and diagnosis has a strong effect on the variance compared to other covariates (Fig. 5b). We identified 140 and 167 differentially expressed genes between control and schizophrenia (referred to as “szDEGs” henceforth) for NeuN^+^ and OLIG2^+^, respectively, at FDR < 0.01 (Fig. 5c; Additional file 1: Tables S8–S9; see the “Methods” section). We compared our results to the previous results obtained from bulk tissues [5, 6] and identified common and distinct sets of differentially expressed genes across the datasets (Additional file 1: Tables S10–S11; see the “Methods” section). Comparing the effect sizes of commonly differentially expressed genes (*P* < 0.05) among the three datasets analyzed, we find significant correlations to the CMC and BrainSeq datasets [5, 6] in NeuN^+^, but not when we compare OLIG2^+^ (Fig. 5d). These results are consistent with the aforementioned deconvolution analysis, indicating that bulk tissue brain studies were limited in terms of non-neuronal signals, such as those coming from oligodendrocytes.

Newly identified szDEGs are enriched for variants for specific disorders or cognitive traits (Fig. 5e; see the “Methods” section). Notably, NeuN^+^ szDEGs are enriched for GWAS signal from schizophrenia and ADHD as well as educational attainment. Interestingly, OLIG2^+^ szDEGs are enriched for genetic variants associated with bipolar disorder and autism spectrum disorders (Fig. 5e), indicating potential cell type-specific relationship between genetic variants and disease-associated variation of gene expression.

Finally, we investigated the relationship between schizophrenia-associated differential DNA methylation and differential gene expression. Remarkably, similar to what we have observed in DNA methylation, szDEGs are preferentially found in genes that are significantly differentially expressed between cell types for both NeuN^+^ (OR = 7.7, FDR = 8 × 10^−8^) and OLIG2^+^ (OR = 13, FDR = 7 × 10^−13^), furthering the functional implication of cell type-specific regulation in schizophrenia. Due to the small number of szDMPs identified at FDR < 0.2, there was little direct overlap between szDMPs and szDEGs. However, when we examined the top 1000 szDMPs, we begin to observe significant enrichments of szDMPs in szDEGs (Fig. 5f). Notably, the top 1000 szDMPs are enriched in genic (3′UTR and exon) and intergenic CpGs in NeuN^+^, while OLIG2^+^ show specific enrichment for intronic and promoter CpGs (Fig. 5f) (Fisher’s exact test, all comparisons FDR < 0.05). These results underscore the promise of cell type-specific approaches to elucidate the relationships between genetic variants, epigenetic modifications, and gene expression in a complex neuropsychiatric disorder.

## Discussion

The etiology of schizophrenia remains largely unresolved even though significant efforts have gone into understanding the genetic and molecular mechanisms of the disease [1]. These efforts have been challenged by both the genetic heterogeneity of the disorder as well as the inherent cellular heterogeneity of the brain. To address these issues, we integrated whole-genome sequencing, transcriptome, and epigenetic profiles from two major cell types in the brain. Whole-genome patterns of DNA methylation and gene expression are highly distinct between cell types, complementing other analyses of cell type-specific epigenetic variation [9, 33]. In particular, our data offer novel resource from oligodendrocytes, a major yet relatively underexplored cell type in the human brains. Indeed, we show evidence that previous analyses of bulk tissue gene expression were underpowered to detect oligodendrocyte-specific signals, underscoring the strength of a cell-specific approach and the fact that most bulk tissue brain studies tend to focus on or specifically isolate gray matter.

A caveat to our study is that methylome and expression studies using human brain tissue could be confounded by the multitude of environmental factors that can impact these measurements such as the use of medications or other drugs, smoking, alcohol use, and other lifestyle factors. We provide such information for the subjects used in this study in Additional file 1: Table S1; however, these data are rarely quantitative and are frequently unknown for many individuals. We therefore compared CpGs previously associated with tobacco smoking [34–36] and did not find a significant overlap with our identified szDMPs (see the “Methods” section). This result suggests that our data are likely not confounded by at least tobacco smoking.

To our knowledge, this is the first study to identify the cell-specific correspondence between whole-genome methylation and expression in brain tissue from patients with schizophrenia. Compared to substantial cell type differences, methylation differences between control and schizophrenia are small. Considering 20% false positives and coverage, we identified 97 szDMPs, compared to over 4 million cell type-specific DMPs identified at a more stringent cutoff of Bonferroni *P* < 0.05. Nevertheless, schizophrenia-associated epigenetic and transcriptomic alteration is highly cell type-specific, thus offering the first direct support to the idea that cell type-specific regulation may be implicated in schizophrenia pathophysiology [9, 33]. Notably, our resource provides novel whole-genome methylation data from affected brain samples rather than making these connections based on genetic associations. By doing so, we demonstrate that cell type epigenetic difference is reduced in affected individuals, offering a potential mechanistic link between dysregulation of cell type-specific epigenetic distinction and disease etiology. The decrease in cell type differences in schizophrenia could be due to a number of pathophysiological mechanisms including a change in cell type differentiation, an alteration in cell type heterogeneity, or a reflection of other unknown altered developmental programs. Patient-derived neurons from iPSCs have not yielded robust observable differences in gene expression [37]. While issues of power have been suggested as the cause of the lack of observable differential expression between cases and controls, it is also plausible that such negative results are due to a critical interplay of multiple nervous system cell types such as oligodendrocytes that are not present in such human culture systems. Future studies that integrate human oligodendrocytes into cellular and other model systems might be able to tease apart the mechanisms by which neuronal and non-neuronal cell types become more similar in schizophrenia. In addition, the use of single-cell methylome and expression profiling in brain tissue from patients should elucidate the spectrum of heterogeneity of cell types in schizophrenia. Recent work has demonstrated that chromatin remodeling in neurons but not astrocytes is relevant to schizophrenia [38]; however, this study did not examine oligodendrocytes. Thus, there are intrinsic molecular differences within each of these major cell classes that can independently be contributing to disease. Based upon our findings, further investigations into the contributions of oligodendrocytes to schizophrenia are warranted.

A large portion of differential DNA methylation between control and schizophrenia occur in non-coding regions. This observation further highlights the role of regulatory variation in disease etiology, similar to the findings from GWAS studies, especially the distribution of schizophrenia genetic risk loci [4, 39, 40]. Notably, the majority of sites that show signals of differential DNA methylation are not accessed by most DNA methylation arrays. Our study demonstrates that schizophrenia pathophysiology is unlikely to be further delineated via the study of differential methylation or expression in the brain given currently used technologies. What we have found is that non-neuronal cells such as oligodendrocytes are just as likely to play a role in disease as neurons. Therefore, the use of emerging technologies to profile individual cells might be able to assess the contribution of even more cell types such as astrocytes or microglia. Moreover, for human brain tissue studies of schizophrenia, we are limited to adult tissues whereas the critical windows of altered methylation and/or expression might be occurring earlier in the development prior to the onset of symptoms and diagnosis. Finally, the heterogeneity of schizophrenia might challenge the interpretation of data from this sample size. Future studies that compare individuals based on specific aspects of disease (e.g., presence of psychosis) might yield greater differences. Nevertheless, what our study has uncovered are a number of cell type changes in expression and methylation that correspond to disease status. In particular, the oligodendrocyte changes are compelling as previous studies were underpowered to detect these changes. With these identified genes in hand, the importance of these specific genes in brain development and function can now be studied in cellular and animal models. These gene lists can also be integrated with future whole-genome sequencing studies.

## Conclusions

We provide the first detailed interrogation of DNA methylation differences between neurons and oligodendrocytes and between brain tissues from patients with schizophrenia compared to controls. These data demonstrate an extensive epigenetic distinction between two major cell types in the brain and that cell type-specific methylation is dysregulated in a specific way in the brains from patients with schizophrenia. These data can be used for prioritizing targets for further experimental analyses. With rapidly decreasing sequencing costs, candidates and hypotheses generated from our study should lead to future analyses at the individual cell level from specific populations of patients (e.g., patients with psychosis or not) to further elucidate the biological alterations associated with schizophrenia.

## Methods

### Sampling strategy

Frozen brain specimens from Brodmann area 46 were obtained from several brain banks (Additional file 1: Tables S1–S2). Cases and controls were matched by age group, and additional demographics such as gender were matched when possible (Additional file 1: Table S1). Information on comorbidities and cause of death when known are included in Additional file 1: Table S1.

### Nuclei isolation from human postmortem brain

Nuclei isolation was performed as described previously [18, 41] with some modifications. Approximately 700 mg of frozen postmortem brain was homogenized with lysis buffer (0.32 M sucrose, 5 mM CaCl_2_, 3 mM Mg(Ac)_2_, 0.1 mM EDTA, 10 mM Tris-HCl pH 8.0, 0.1 mM PMSF, 0.1% (w/o) Triton X-100, 0.1% (w/o) NP-40, protease inhibitors (1:100) (#P8340, Sigma, St. Louis, MO), RNase inhibitors (1:200) (#AM2696, ThermoFisher, Waltham, MA)) using a Dounce homogenizer. Brain lysate was placed on a sucrose solution (1.8 M sucrose, 3 mM Mg(Ac)_2_, 10 mM Tris-HCl pH 8.0) to create a concentration gradient. After ultracentrifuge at 24,400 rpm for 2.5 h at 4 °C, the upper layer of the supernatant was collected as the cytoplasmic fraction. The pellet, which included the nuclei, was resuspended with ice-cold PBS containing RNase inhibitors and incubated with mouse alexa488 conjugated anti-NeuN (1:200) (#MAB377X, Millipore, Billerica, MA) and rabbit alexa555-conjugated anti-OLIG2 (1:75) (#AB9610-AF555, Millipore) antibodies with 0.5% BSA for 45 min at 4 °C. Immuno-labeled nuclei were collected as NeuN-positive or OLIG2-positive populations by fluorescence-activated nuclei sorting (FANS). After sorting, gDNA and total RNA were purified from each nuclei population using a ZR-Duet DNA/RNA MiniPrep (Plus) kit (#D7003, Zymo Research, Irvine, CA) according to the manufacturer’s instruction. Total RNA was treated with DNase I after separation from gDNA. Two hundred nanograms total RNA from each sample was treated for ribosomal RNA removal using the Low Input RiboMinus Eukaryote System v2 (#A15027, ThermoFisher) according to the manufacturer’s instruction. After these purification steps, gDNA and total RNA were quantified by Qubit dsDNA HS (#Q32851, ThermoFisher) and RNA HS assay (#Q32852, ThermoFisher) kits, respectively. Immunostaining was visualized using a Zeiss LSM 880 with Airyscan confocal laser scanning microscope. One hundred microliters of sorted nuclei was placed onto microscope slides, and 300 μl of ProLong Diamond Antifade Mountant with DAPI (#P36971, ThermoFisher) was added and covered with glass coverslips before imaging.

### Whole-genome bisulfite sequencing library generation and data processing

As a control for bisulfite conversion, 10 ng of unmethylated lambda phage DNA (#D1501, Promega) was added to the 1 μg of input DNA. Libraries were made with an in-house Illumina sequencer-compatible protocol. The extracted DNA was fragmented by S-series Focused-ultrasonicator (Covaris, Woburn, MA) using the “200-bp target peak size protocol.” Fragmented DNA was then size selected (200–600 bp) with an Agencourt AMPure XP bead-based (#A63880, Beckman Coulter, Brea, CA) size selection protocol [42]. The DNA end repair step was performed with End-It DNA End-Repair Kit (#ER81050, Epicentre, Madison, WI). After the end-repair step, A-tailing (#M0202, New England Biolabs, Ipswich, MA) and ligation steps were performed to ligate the methylated adaptors.

Bisulfite treatment of gDNA was performed using the MethylCode Bisulfite Conversion Kit (#MECOV50, ThermoFisher). Purified gDNA was treated with CT conversion reagent in a thermocycler for 10 min at 98 °C, followed by 2.5 h at 640 °C. Bisulfite-treated DNA fragments remain single-stranded as they are no longer complementary. Low-cycle (4–8) PCR amplification was performed with Kapa HiFi Uracil Hotstart polymerase enzyme (#KK2801, KAPA Biosystems, Wilmington, MA) which can tolerate uracil residues. The final library fragments contain thymines and cytosines in place of the original unmethylated cytosine and methylated cytosines, respectively.

The methylome libraries were diluted and loaded onto an Illumina HiSeq 2500 or HiSeqX system for sequencing using 150 bp paired-end reads. We generated over 900 million reads per sample and performed quality and adapter trimming using TrimGalore v.0.4.1 (Babraham Institute) with default parameters. Reads were mapped first to the PhiX genome to remove the spike-in control, and the remaining reads were mapped to the human GRCh37 (build 37.3) reference genome using Bismark v 0.14.5 [43] and bowtie v1.1.2 [44]. We removed reads with exact start and end positions using Bismkar deduplication script. After de-duplication, we calculated the fractional methylation levels at individual cytosines [32]. Overall, we generated a total of 72.6 billion reads (equivalent to 10.9 T base pairs of raw sequence data) and obtained per-sample average coverage depths > 25× covering 98% of the 28 million CpGs in the human genome (Additional file 1: Table S12). Bisulfite conversion rates were estimated by mapping the reads to the lambda phage genome (NC_001416.1), see Additional file3: Figure S13 for a general overview of the WGBS data quality and processing.

### Whole-genome sequencing data processing

Quality and adapter trimming was performed using TrimGalore v.0.4.1 (Babraham Institute) with default parameters. Reads were mapped to the human GRCh37 reference genome using BWA v0.7.4 [45], and duplicates were removed using picard v2.8.3 (https://broadinstitute.github.io/picard/index.html). We identified genetic polymorphisms from re-sequencing data following GATK v3.7 best practices workflow [46]. Specifically, we used HapMap 3.3, Omni 2.5 M, 1000 Genomes Phase I, and dbSNP 138 as training datasets for variant recalibration. We filtered variant calls with high genotype quality (GQ ≥ 20.0). Overall, we generated a total of 225 million reads and identified 15,331,100 SNPs with mean depth above > 16.5× (Additional file 1: Table S13). We removed the polymorphic cytosines from downstream differential methylation analyses keeping a total of 24,942,405 autosomal CpGs (Additional file 1: Table S14), see Additional file 3: Figure S13 for a general overview of the WGS data quality and processing.

For quality control of the SNP calling, we performed principal component analyses using an additional 210 samples from 4 different populations from the HapMap Project (60 CEU, 90 CBH/JPT, and 60 YRI) to explore the genetic ancestry of the individuals. After LD pruning (*r*^2^ > 0.2) with SNPRelate R package, we used 66,667 autosomal polymorphic SNPs in the analysis. The PC plot shows that the reported ancestry of the individuals was mostly concordant to that inferred from the SNPs (Additional file 3: Figure S14), validating the genotype calling. The first 10 genetic PCs were included in the differential methylation analyses to control for population structure (Additional file 1: Table S14).

### Hierarchical clustering of methylomes from diverse human cell types

We added WGBS data from additional tissues [12] (see original references for the datasets therein) and Lister et al. [27], and the corresponding genome coordinates (hg38 and hg18) were converted to hg19 using UCSC Batch Coordinate Conversion tool (liftOver executable) [47]. The sample indicated with the star in Fig. 2a was also remapped to hg38 from raw data following the same protocol as other non-brain tissues (from Mendizabal and Yi [12]) and lifted over to hg19. The clustering of the two methylomes from the same individual “NeuN+_ind2” suggests no significant effect of mapping/lift over in the clustering results. A total of 14,115,607 CpG positions with at least 5× coverage in all individuals were used to draw a hierarchical clustering tree (using R stats package’s hclust function with method = average (= UPGMA) based on Euclidean distances using fractional methylation values using dist function). The tree was plotted using dendextend and circlize packages.

### Identification of differentially methylated positions and regions between OLIG2^+^ and NeuN^+^

We identified DMPs between 25 NeuN^+^ and 20 OLIG2^+^ individuals by using DSS [29]. DSS handles variance across biological replicates as well as model read counts from WGBS experiments. Importantly, DSS also considers other biological covariates that may affect DNA methylation patterns. Specifically, we considered age, gender, brain hemisphere, postmortem interval (PMI), conversion rates, brain bank, and genetic ancestry (using the first 10 genetic PCs obtained from WGS of the same individuals) as covariates (Additional file 1: Tables S1–S2 and S14; Additional file 3: Figure S15). Age and PMI were converted to categorical variables (“AgeClass” and “PMIClass” in Additional file 1: Table S2).

Since C>T and G>A polymorphisms at CpGs could generate spurious differentially methylated sites on bisulfite conversion experiments, we excluded polymorphic CpGs (identified from re-sequencing the same panel of individuals, Additional file 1: Table S15) from DMP analyses. For DMP identification between OLIG2^+^ and NeuN^+^ samples, we used a Bonferroni cutoff on *P* < 0.05 and identified 4,058,898 DMPs out of 24,596,850 CpGs tested. For DMR identification, we considered a minimum region of 50 bp with at least 5 significant DMPs and identified 145,073 regions (Additional file 2: Table S3). We explored the effect of coverage on cell type DMP identification and found that low-coverage sites had a limited contribution to the significant DMPs; indeed, relatively more sites were detected at more stringent coverage thresholds. For example, removing sites < 5× in 80% of individuals within each cell type led to a total of 4,037,979 significant DMPs at Bonferroni 0.05 cutoff (out of 23,788,847 CpGs, 16.97%), whereas the removal of sites < 10× lead to 3,903,652 DMPs (out of 21,399,153 CpGs tested, 18.2%), and < 20× lead to 2,509,489 DMPs (out of 10,960,268 CpGs considered, 23.8%). Enrichments between cell type DMPs and szDMP and between cell type DMPs and ChIP-seq peaks were similar when using the > 20× coverage datasets instead of using all sites.

Of note, as our differential methylation analyses are run under a multifactor design in DSS, the estimated coefficients in the regression are based on a generalized linear model framework using arcsine link function to reduce the dependence of variance on the fractional methylation levels [29, 48]. Thus, whereas the direction of change is indicated by the sign of the test statistic, its values cannot be interpreted directly as fractional methylation level differences. The distribution of the statistic depends on the differences in methylation levels and biological variations, as well as technical factors such as coverage depth. For DMRs, the method provides “areaStat” values which are defined as the sum of the test statistic of all CpG sites within the DMR. To obtain a more interpretable estimate of fractional methylation differences, we also provide results for a linear model using the same formula as for DSS.

### Functional characterization of DMRs

For different enrichment analyses, we generated matched control regions. We generated 100 sets of regions with similar genomic properties as the DMRs: number of total regions, region length distribution, chromosome, and matched GC content within 1%. Empirical *P* values were computed by counting the number of matched control sets showing values as extreme as the observed one. Enrichments were computed as the ratio between the observed value and the mean of the matched control sets. We used ChIPSeeker [49] and bioconductor’s UCSC gene annotation library TxDb.Hsapiens.UCSC.hg19.knownGene to annotate DMRs to genes. We explored the 25 chromatin state model maps based on ChIP-Seq experiments on 6 chromatin marks (H3K4me3, H3K4me1, H3K36me3, H3K27me3, H3K9me3, and H3K27ac) from the Roadmap Epigenomics Project [28]. We joined several categories related to enhancer states, including TxReg, TxEnh5’, TxEnh3’, TxEnhW, EnhA1, EnhA2, EnhW1, EnhW2, and EnhAc.

### Overlap with neuronal and non-neuronal ChIP-seq datasets

We analyzed the overlap between our cell type-specific DMPs and DMRs with neuron and non-neuron histone mark data on H3K4me3 and H3k27ac ChIP-seq experiments [9]. We only considered peaks that were assigned as “neuronal” and “non-neuronal” and discarded “NS” peaks from Additional file 1: Table S11 in the cited paper. To test directionality with our OLIG2^+^ vs. NeuN^+^ differentially methylated sites, we further discarded peaks that overlapped between cell types (i.e., neuronal H3K4me3 peaks overlapping with non-neuronal H3K27ac, and non-neuronal H3K4me3 peaks overlapping with neuronal H3K27ac peaks).

### Non-CpG methylation patterns in brain cell types

We studied DNA methylation patterns of NeuN^+^ and OLIG2^+^ outside CpG dinucleotides (CH context). Given the low fractional patterns of DNA methylation outside CpG sites, and to minimize the influence of any additional covariates, only individuals with conversion rates ≥ 0.995 were considered (15 NeuN^+^ and 14 OLIG2^+^). We filtered cytosines that showed less than 5× coverage in 90% of individuals per cell type, as well as removed the positions with genetic polymorphisms (C>T and T>C SNPs to account for SNPs at both strands). A total of 333 and 457 million cytosines remained in NeuN^+^ and OLIG2^+^, respectively. Cytosines in gene bodies were filtered using BEDtools [50].

### Identification of DMPs between schizophrenia and control individuals

We used DSS to identify DMPs between schizophrenia and control samples. Again, we considered biological covariates in the differential methylation analyses, namely age, gender, brain hemisphere, PMI, conversion rates, brain bank, and genetic ancestry (using the first 10 genetic PCs obtained from WGS of the same individuals, see File S3 for specific commands used). For an FDR cutoff of 0.2 for significance, we identified a total of 201 and 60 DMPs in OLIG2^+^ and NeuN^+^, respectively. We further filtered sites with less than 20× in at < 80% of individuals per group. We identified 14 and 83 significant DMPs in NeuN^+^ and OLIG2^+^, respectively, when applying a FDR < 0.2.

As a comparison, we also ran differential methylation analyses for disease using a linear model based on fractional methylation values for every CpGs site and considered the same covariates as in the DSS analyses. We plotted quantile-quantile plots for the expected and observed *P* values obtained from DSS and linear model analyses between schizophrenia and control, as well as to evaluate how coverage affects these two methods. We observed that DSS provides correction for low-coverage sites, note the systematic depletion of good *P* values at low-coverage sites in DSS (Additional file 3: Figure S16), compared to high-coverage sites. In contrast, a linear model shows a similar genome-wide distribution of *P* values at low- and high-coverage sites. We identified a total of 60 and 210 CpGs in NeuN^+^ and OLIG2^+^, respectively, at FDR < 0.2. However, to obtain a more conservative set of hits, we additionally filter for high-coverage sites (20× in at least 80% of samples per disease-control group) and recalculated FDR, obtaining 14 and 83 significant sites at FDR < 0.2. In order to test the robustness of the results and the effect of covariates as well as the potential hidden structures in the data, we performed a permuting analysis by randomly assigning case/control labels and re-ran DSS 100 times.

### Power analyses for DMP identification between schizophrenia and control individuals

In this first power analysis, we determined the range of effect sizes that can be detected at different *P* value thresholds in our genome-wide scan focused on detecting individual DMPs. The main aim of this analysis was to determine the power of our study to detect individual DMPs at different significance thresholds, using realistic parameters that mimic the fractional methylation values seen in cases and controls. Specifically, we simulated 10 million CpGs following these steps:

1. In the first step, for each simulated CpG, we sample the parametric mean of fractional methylation values in controls from a truncated normal distribution (mirroring the skew in genome-wide fractional methylation values): rtnorm(simulations,0.7,0.05, lower = 0.1,upper = 0.9)
2. We next obtain the parametric standard deviation (SD) of fractional methylation values for the CpG in controls (by sampling from a uniform distribution that mimics the genome-wide distribution of SD seen in our data): runif(simulations,0.0000001,0.2)
3. After having determined the parametric mean and SD in controls, we used these to obtain the fractional methylation values in as many simulated control individuals as we used in our study (*n* = 25 as in the NeuN analysis). rtnorm(control.sample.size, control.mean, control.sd, lower = 0,upper = 1)
4. We next select a case-control difference value (effect size, or parametric *β*) at each simulated CpG by drawing values from a uniform distribution. runif(simulations,0,0.20).
5. After obtaining the effect size at each simulated CpG, the mean fractional methylation value in cases can be obtained by adding the case-control difference (from step 4) to the control mean methylation values (step 3). Then, we sample the number of cases from a truncated normal distribution using the mean of cases and the standard deviation for cases (same as for controls, as we do not observe differences in SD in the real data between the groups).
6. rtnorm(case.sample.size, case.mean, case.sd, lower = 0,upper = 1)
7. We perform a linear regression of case/control labels on methylation. lm(methylation~diagnosis)

Additional file 3: Figure S7a shows the heatmap of the average power for the full grid of parameters used to simulate the 10 million DMPs (CpGs that present differential methylation between the simulated cases and controls). The population effect sizes (absolute case-control differences) and the *P* value achieved at each simulated DMP are shown in the *X*-axis and *Y*-axis, respectively.

We extract two important conclusions from the heatmap figure. First, our study is certainly not particularly well-powered to detect small differences in average fractional methylation values between cases and controls. For instance, less than 20% of DMP effects in the range of 1 to 4% achieve *P* < 10^−5^ to *P* < 10^−7^ in our simulated study (blue vertical band at the left side of the heatmap). It is important to note that the total number of such effects in schizophrenia remains unknown; however, it is certainly possible given the polygenic nature of schizophrenia observed in most *omics* datasets [5, 39]. Therefore, an apparently low positive power (10 to 20%) may still imply that hundreds of genome-wide real effects achieve approximately *P* < 10^−5^ in our study.

The second implication of this analysis carries a more positive message in regard to the power of a genome-wide with the sample size from our study. Specifically, starting from 5% differences in average, a large fraction (about a third) of simulated DMPs pass a significance threshold of *P* < 10^–5^, and ~ 50% of those with effects > 8% achieve *P* < 10^−5^ and deeper significance thresholds. Notably, these are precisely the range of effects that we report at the *P* value cutoffs that correspond to the FDR 20% we use in our study (*P* values ranging from 3.6 × 10^−7^ to 8.54 × 10^−9^ in NeuN^+^ and 1.36 × 10^−6^ to 8.18 × 10^**−**14^ in OLIG2^+^), being the effect size around 6.4% in average (ranging from 3.3 to 12.8% in NeuN^+^ and from 1.12 to 22.4% in OLIG2^+^).

As mentioned above, the balance between true and false positives at different *P* value thresholds depends on the underlying (and currently unknown) distribution of effect sizes of DMPs and the total number of them that are present genome-wide. For this reason, in our genome-wide scan, we favored a strict control of multiple testing to avoid the detection of false effects. Still, akin to the first generation of GWAS and as shown by the robust departure from the random expectation shown by the quantile-quantile plots, we report in Fig. 2a a large fraction of our top signals are likely true positives.

We would like to note here that previously obtained effect sizes for schizophrenia-associated CpGs in brain samples were generally small, for instance, around 1.48% (ranging from 0.41 to 4.42%, in Jaffe et al. [7]). However, these estimates correspond to the analyses based on methylation profiling of bulk tissue and focusing on a more limited set of the CpGs available genome-wide (~ 0.4 million CpGs). If schizophrenia-associated CpG sites showed cell type-specific patterns and/or were located outside the targeted CpGs in methylation array chips, these effect sizes could be underestimates of the actual case/control differences. Thus, unbiased whole-genome scanning of 25 million CpGs in purified cell types could potentially identify bigger case/control differences, and the sample sizes we present in this study would be moderately empowered.

In summary, this first power simulation study suggests that even with our small sample sizes, we can detect CpGs with moderate-to-large effect sizes. Although less powered to detect the bulk of small effects (~ 0.01 differences), this should not offset the inherent interest of a first genome-wide study that spans millions of CpGs in purified cell types, since we are powered to detect effects that would not be detected in previous case-control attempts for schizophrenia. Importantly, most of these sites appear in regions currently not included in widely used methylation arrays.

In the second power analysis, we explored realistic parameters in regard to the total number of differentially methylated DMPs and the true distribution of effect sizes between cases and controls, in order to make robust inferences into the lists of DMPs and effect sizes that would make it into the top 1000 list of most significant effects. To obtain estimates of the true- and false-positive rates in the top 1000 szDMPs, we first need to obtain plausible genetic architectures of methylation differences in schizophrenia (i.e., the total number of real DMPs, and their effect sizes). According to genome-wide association studies, schizophrenia is a polygenic disease in which each variant exerts a small effect on the phenotype [39]. Thus, we assumed in our analyses that the epigenetic architecture for schizophrenia follows a similar pattern.

Specifically, we modeled the real distribution of effect sizes in our simulations using a beta distribution that permits to assign values between 0 and 1. This probability distribution is parameterized by two shape parameters, denoted as *α* and *β* (also referred to as parameters 1 and 2 here). Assuming 5000 DMPs (CpGs with differential methylation between schizophrenia cases and healthy controls), we explored a range of effect size distributions obtained by the two parameters. Specifically, we explored [0.1,1.5] and [20,51] for each parameter, as these are the ranges that give long-tailed distributions of effect sizes with a peak at 0.01 to 0.1 and a maximum DMP effect of ~ 0.4 (40%).

In each simulation (*n* = 50,000), after obtaining the distribution of effect sizes of the 5000 causal DMPs, we performed a genome-wide scan with all 10 million CpG (*P* values of non-causal CpGs are obtained from a uniform distribution [0,1]). We then ranked the 10 million sites per *P* value and checked how many of the causal CpGs make it in the top 1000 values. As shown in Additional file 3: Figure S7b, we found the area that yielded 0.5 of FDR at the top 1000 szDMPs, as we observe in our data.

Using the range of parameter 1 and parameter 2 values that give FDRs around 50% (the green band in Additional file 3: Figure S7b), we then asked which case-control differences and *P* values are observed at the true szDMPs found at the top 1000 loci. As shown in the histogram plot in Additional file 3: Figure S7c, we find that the effect sizes of true szDMPs are indeed substantial. Of note, the best 1000 *P* values in the szDMPs per cell type observed in our study show an average of 4.85% case/control difference at the following *P* value thresholds: 7.31 × 10^−5^ in NeuN^+^ and 4.16 × 10^−5^ in OLIG2^+^.

In summary, this second simulation study shows that even though the top 1000 CpGs certainly contain a fraction of false positives (~ 50% as measured by the FDR corresponding to the 1000th CpG in our study), the other ~ 50% of CpGs consist of true positives enriched for moderate-to-large effect sizes. This enrichment justifies using this relaxed set of loci to obtain some biological insights given the restricted loci with FDR < 0.2.

### szDMP gene annotation and functional enrichment

We used ChIPSeeker [49] and bioconductor’s UCSC gene annotation library TxDb.Hsapiens.UCSC.hg19.knownGene to annotate the top 1000 szDMPs to genes (ordered by *P* values). We used genes associated with genic szDMPs only (all annotation categories excluding distal intergenic, defined as > 1.5 kb from the start or end of genes) for functional enrichment using ToppGene [52]. We also explored the potential of szDMPs to bind transcription factors by intersecting the top 1000 szDMPs (ordered by *P* value) from each cell type with the ENCODE transcription factor ChIP-Seq datasets. We downloaded the “wgEncodeRegTfbsClusteredV3” table from UCSC and counted the number of szDMPs showing TF binding. We compared these numbers to 100 sets of random 1000 CpGs with large *P* values for schizophrenia-control comparison (*P* > 0.1). We also calculated the enrichment of specific transcription factors by comparing the frequency of each of the 161 transcription factors between szDMPs and non-szDMPs. The enrichments were obtained by dividing the observed number to the average of 100 sets, and the *P* values show the number of times the number for szDMPs was larger than the 100 sets.

### szDMP enrichment at GWAS

Genome-wide *P* values and odds ratios for GWAS for schizophrenia [4], smoking [53], clozapine-induced agranulocytosis [54], coronary artery disease, bipolar disorder [51], autism spectrum disorder, and anorexia nervosa were downloaded from the Psychiatric Genomics Consortium at https://www.med.unc.edu/pgc/results-and-downloads/. Data for rheumatoid arthritis [55] were downloaded from ftp://ftp.broadinstitute.org/pub/rheumatoid_arthritis/Stahl_etal_2010NG/. In order to explore the potential contribution and/or mediation of DNA methylation to the genetic basis of schizophrenia, we explored the co-localization of the top 1000 szDMPs with GWAS results. Given that the majority of the schizophrenia heritability is found below the significance thresholds of GWAS, we explored the patterns at genome-wide SNPs as follows. For each szDMP, we identified all SNPs reported by the GWAS study within a 1-kb window and counted the number of SNPs at different quantiles of odds ratio (OR). We used quantiles of OR so that we can compare the different diseases and traits among them. We repeated this step using the same number of random non-szDMPs 100 times. To obtain empirical *P* values, we calculated the number of times non-szDMP sets showed more SNPs in each OR quantile than szDMPs. SNPs with moderate-to-high OR in schizophrenia GWAS consistently showed low empirical *P* values for both cell type DMPs, implying that SNPs with large effect sizes in GWAS studies are closer to szDMPs than expected. Interestingly, this pattern was not observed for other traits, implying the co-localization is exclusive to the disease.

### Hydroxymethylation at szDMPs

We compared our results to a single-base resolution hydroxymethylome maps [56]. Specifically, TAB-seq data from an adult human brain sample was obtained from GEO (GSE46710). We used the sites presenting high hmC as defined in the original paper (hmC > mC; *n* = 5,692,354). We plotted quantile-quantile plots of DSS statistic *P* values at high hmC loci and random loci. These analyses showed no significant presence of hmC in the szDMPs (Additional file 3: Figure S17).

### Smoking DMPs at szDMP

We explored the co-localization of szDMPs with CpGs associated with tobacco smoking [34–36]. None of the analyzed smoking DMPs (*n* = 206) was found among our szDMPs at FDR < 0.2 nor at the top 1000 CpGs with best *P* values per cell type. These analyses suggest that szDMPs might not be confounded by smoking.

### Targeted validation experiments

We designed high-coverage bisulfite experiments to sequence 18 regions (Additional file 1: Table S16) from 44 samples (including 24 new individuals not included in the WGBS experiments, Additional file 1: Table S17). We conducted bisulfite conversions of gDNA from OLIG2^+^ and NeuN^+^ cells using EZ DNA Methylation-Gold Kit (#D5006, Zymo Research) according to the manufacturer’s instructions. Sodium bisulfite converted unmethylated cytosines to uracil while methylated cytosines remained unconverted. Upon subsequent PCR amplification, uracil was ultimately converted to thymine. Bisulfite sequencing PCR primers were designed using MethPrimer 2.0 and BiSearch to target a panel of 12 loci in OLIG2^+^ and 6 loci in NeuN^+^ (Additional file 1: Table S16). The primers were designed with an Illumina adaptor overhang. The sites of interest were amplified using JumpStart Taq DNA polymerase (#D9307, Sigma) and quantified using gel electrophoresis to verify the size and Qubit fluorometric quantitation to determine the concentration. Equimolar quantities of each of the target amplicons were pooled for each individual, and NGS libraries were prepared in a second PCR reaction according to Nextera XT DNA Sample Preparation protocol. The libraries were barcoded with a unique pair of Nextera XT primers. The libraries were sequenced with Illumina MiSeq using the 500-cycle kit (250 paired-end sequencing). We sequenced the samples at high coverage using a MiSeq machine and 250 bp paired-end reads at the Georgia Institute of Technology High Throughput DNA Sequencing Core. We mapped the reads to the human GRCh37 (build 37.3) reference genome using Bismark v0.20.2 and Bowtie v2.3.4. We trimmed the reads for low quality and adapters using TrimGalore v.0.5.0 (Babraham Institute) with default parameters. Only the sites with at least 200× coverage were considered (mean = 14,580, median = 10,810). One region showed low read counts and was excluded (Additional file 1: Table S16). A total of 16 DMPs and an additional 50 adjacent CpGs were considered in the validation analyses. Fractional methylation values were adjusted for covariates using the following linear model: lm (methylation ~ diagnosis + sex + age_class + PMI_class).

### Concordance with previous methylation studies on schizophrenia

We evaluated the concordance between our disease DMP signals with Jaffe et al. [7] which used bulk brain tissue and Illumina 450 K chips. We binned Jaffe et al. study’s whole-genome *P* values and calculated the fraction of CpGs in our study showing the same directionality in both studies (i.e., hypomethylated or hypermethylated in disease vs. control). For each cell type, we tested the significance at each *P* value bin using a Binomial test with *P* = 0.5 expectation. We additionally compared the distribution of concordance rates from the 100 control datasets obtained using case/control permuted labels and re-running DSS on them.

### RNA sequencing

RNA-seq was performed as described previously [57]. Total RNA from the cytoplasmic fraction was extracted with the miRNeasy Mini kit (#217004, Qiagen, Hilden, Germany) according to the manufacturer’s instruction. The RNA integrity number (RIN) of total RNA was quantified by Agilent 2100 Bioanalyzer using Agilent RNA 6000 Nano Kit (#5067-1511, Agilent, Santa Clara, CA). Total RNAs with an average RIN value of 7.5 ± 0.16 were used for RNA-seq library preparation. Fifty nanograms of total RNA after rRNA removal was subjected to fragmentation, first and second strand syntheses, and clean up by EpiNext beads (#P1063, EpiGentek, Farmingdale, NY). Second-strand cDNA was adenylated, ligated, and cleaned up twice by EpiNext beads. cDNA libraries were amplified by PCR and cleaned up twice by EpiNext beads. cDNA library quality was quantified by a 2100 Bioanalyzer using an Agilent High Sensitivity DNA Kit (#5067-4626, Agilent). Barcoded libraries were pooled and underwent 75 bp single-end sequencing on an Illumina NextSeq 500.

### RNA-seq mapping, QC, and expression quantification

Reads were aligned to the human hg19 (GRCh37) reference genome using STAR 2.5.2b [58] with the following parameters: *--outFilterMultimapNmax 10 --alignSJoverhangMin 10 --alignSJDBoverhangMin 1 --outFilterMismatchNmax 3 --twopassMode Basic*. Ensemble annotation for hg19 (version GRCh37.87) was used as a reference to build STAR indexes and alignment annotation. For each sample, a BAM file including mapped and unmapped reads with spanning splice junctions was produced. Secondary alignment and multi-mapped reads were further removed using in-house scripts. Only uniquely mapped reads were retained for further analyses. Quality control metrics were performed using RseqQC using the hg19 gene model provided [59]. These steps include: number of reads after multiple-step filtering, ribosomal RNA reads depletion, and defining reads mapped to exons, UTRs, and intronic regions. Picard tool was implemented to refine the QC metrics (http://broadinstitute.github.io/picard/). Gene-level expression was calculated using HTseq version 0.9.1 using intersection-strict mode by exons [60]. Counts were calculated based on protein-coding gene annotation from the Ensemble GRCh37.87 annotation file, see quality control metrics in Additional file 3: Figures S18–S19 and Additional file 1: Table S18.

### Covariate adjustment and differential expression

Counts were normalized using counts per million reads (CPM). Genes with no reads in either schizophrenia (SZ) or control (CTL) samples were removed. Normalized data were assessed for effects from known biological covariates (diagnosis, age, gender, hemisphere), technical variables related to sample processing (RIN, brain bank, PMI), and technical variables related to surrogate variation (SV) (Additional file 3: Figure S20). SVs were calculated using SVA [61] based on “be” method with 100 iterations. The data were adjusted for technical covariates using a linear model:

$$ \mathrm{lm}\left(\mathrm{gene}\ \mathrm{expression}\sim \mathrm{ageclass}+\mathrm{gender}+\mathrm{hemisphere}+\mathrm{PMIClass}+\mathrm{RIN}+\mathrm{BrainBank}+\mathrm{nSVs}\right) $$

Adjusted CPM values were used for co-expression analysis and visualization. For differential expression, we used the lmTest (“robust”) and ebayes functions in the limma [62] fitting all of the statistical models to estimate log_2_ fold changes, *P* values, and FDR/Bonferroni correction. This method was used for (1) cell type differences (|log_2_(fold change)| > 0.5 and Bonferroni FDR < 0.05), (2) NeuN^+^ SZ-CTL analysis (|log_2_(fold change)| > 0.3 and FDR < 0.01), and (3) OLIG2^+^ SZ-CTL analysis (|log_2_(fold change)| > 0.3 and FDR < 0.01). Bonferroni was used in 1 to provide higher stringency on the data analysis.

### Cross-validation

Cross-validation analyses were applied to ensure the robustness of the DEG analysis:

1. Permutation method based on gene expression randomization (nPerm = 200).
2. Leave-one-out method based on subsampling the data (nLOO = 200).

### Functional gene annotation

The functional annotation of differentially expressed and co-expressed genes was performed using ToppGene [52]. A Benjamini-Hochberg FDR (*P* < 0.05) was applied as a multiple comparisons adjustment.

### GWAS data and enrichment

We manually compiled a set of GWAS studies for several neuropsychiatric disorders, cognitive traits, and non-brain disorders/traits. Summary statistics from the genetic data were downloaded from Psychiatric Genomics Consortium (http://www.med.unc.edu/pgc/results-and-downloads) and GIANT consortium (https://portals.broadinstitute.org/collaboration/giant/). Gene-level analysis was performed using MAGMA [63] v1.04, which considers linkage disequilibrium between SNPs. 1000 Genomes (EU) dataset was used as a reference for linkage disequilibrium. SNP annotation was based on the hg19 genome annotation (gencode.v19.annotation.gtf). MAGMA statistics and –log10(FDR) are reported in Additional file 1: Table S19 for each of the GWAS data analyzed. Brain GWAS: *ADHD*, attention deficit hyperactivity disorder [64]; *ASD*, autism spectrum disorders (https://www.biorxiv.org/content/early/2017/11/27/224774); *BIP*, bipolar disorder [65]; *ALZ*, Alzheimer’s disease [66]; *MDD*, major depressive disorder [67]; *SZ*, schizophrenia [4, 65]. Cognitive traits GWAS: CognFun = cognitive function [64], EduAtt = educational attainment [68], Intelligence = intelligence [69]. Non-brain GWAS: *BMI*, body mass index [70]; *CAD*, coronary artery disease [71]; *DIAB*, diabetes [72]; *HGT*, height (https://www.biorxiv.org/content/early/2018/07/09/355057); *OSTEO*, osteoporosis [73].

### Cell type enrichment and deconvolution analyses

MTG single-nuclei RNA-seq was downloaded from Allen Brain Institute web portal [74]. Normalized data and cluster annotation were used to define cell markers using FindAllMarkers in Seurat [75] with the following parameters: logfc.threshold = 0.25, test.use = “wilcox”, min.pct = 0.25, only.pos = TRUE, return.thresh = 0.01, min.cells.gene = 3, and min.cells.group = 3. Enrichment analyses were performed using Fisher’s exact test. Cell type deconvolution was performed using MuSiC [76] with the following parameters: iter.max = 1000, nu = 1e-10, eps = 0.01, and normalize = F.

### Public data analyses

GTEx tissue expression was downloaded from the GTEx web portal. Raw data was normalized using log_2_(CPM + 1) [77]. Gene expression data from SZ and healthy CTL brain tissue was downloaded from the Common Mind Consortium [5]. Gene expression data from SZ and healthy CTL developmental brain tissue was downloaded from Brain Phase1 [6]. We applied differential expression analysis using the lmTest (“robust”) and ebayes functions in the limma [62] fitting all of the technical/biological covariates and surrogate variables to estimate log2 fold changes, *P* values, and FDR/Bonferroni correction. Surrogate variables were calculated with SVA package [61].

## Additional files

Additional file 1: Supplementary Tables S1, S2, S4–S19. (XLSX 3715 kb)

Additional file 2: Supplementary Table S3. (XLSX 18753 kb)

Additional file 3: Supplementary Figures S1–S20. (PDF 1643 kb)

## Data Availability

The datasets generated during the current study have been deposited in the NCBI Gene Expression Omnibus and are accessible through GEO Series accession number GSE108066 at https://www.ncbi.nlm.nih.gov/geo/query/acc.cgi?acc=GSE108066 [78]. Codes to support the analyses and shiny app for expression data visualizations are available at GitHub at https://github.com/konopkalab/Schizophrenia_CellType [79] and https://github.com/soojinyilab/Schizophrenia_CellType and at Zenodo 10.5281/zenodo.3251942 [80] under the GPL General Public License V3.0. Genome-wide DSS results for cell type and disease analyses are available at https://figshare.com/s/7835f68d21874a9f7e09 [81].
