# Supplementary material for: Cell type-specific epigenetic links to schizophrenia risk in the brain
Source: Genome Biol. 2019 Jul 9;20:135. doi: 10.1186/s13059-019-1747-7 (PMC6617737; doi:10.1186/s13059-019-1747-7)

Supplementary Figures

Fig. S1. Enhancer chromatin signatures at cell-type DMRs. A. Number of DMRs showing enhancer-like chromatin states in 127 samples from RoadMap epigenomic maps. **B.** Genic annotation of DMRs that show enhancer chromatin states in brain tissues.

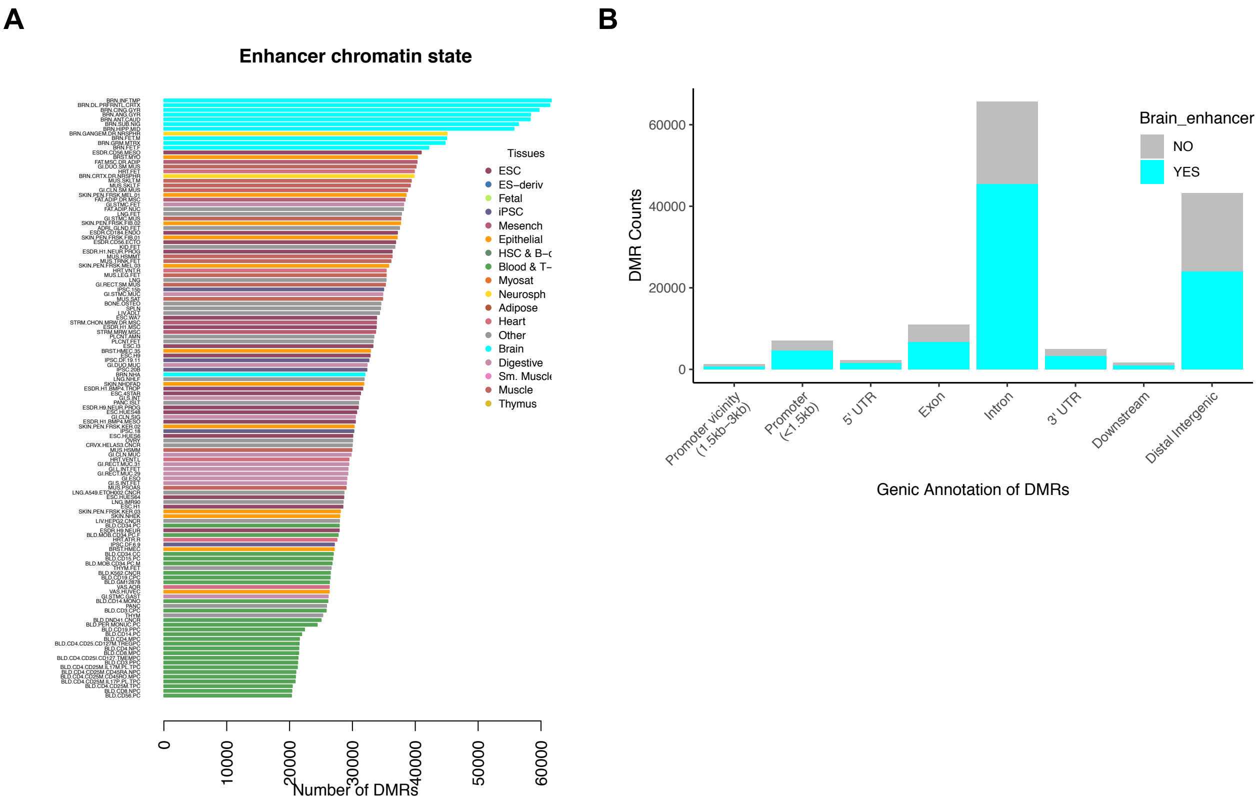

Fig. S2. Distribution of cell-type specific methylation differences at CpGs within H3K4me3 ChIP-seq peaks in NeuN$^+$ and NeuN$^-$ nuclei. Positive values of DSS statistic indicate hypomethylation in NeuN$^+$ compared to OLIG2$^+$ (left), whereas negative values indicate hypomethylation in OLIG2$^+$ (right). Dashed lines indicate the significance level for DSS analyses.

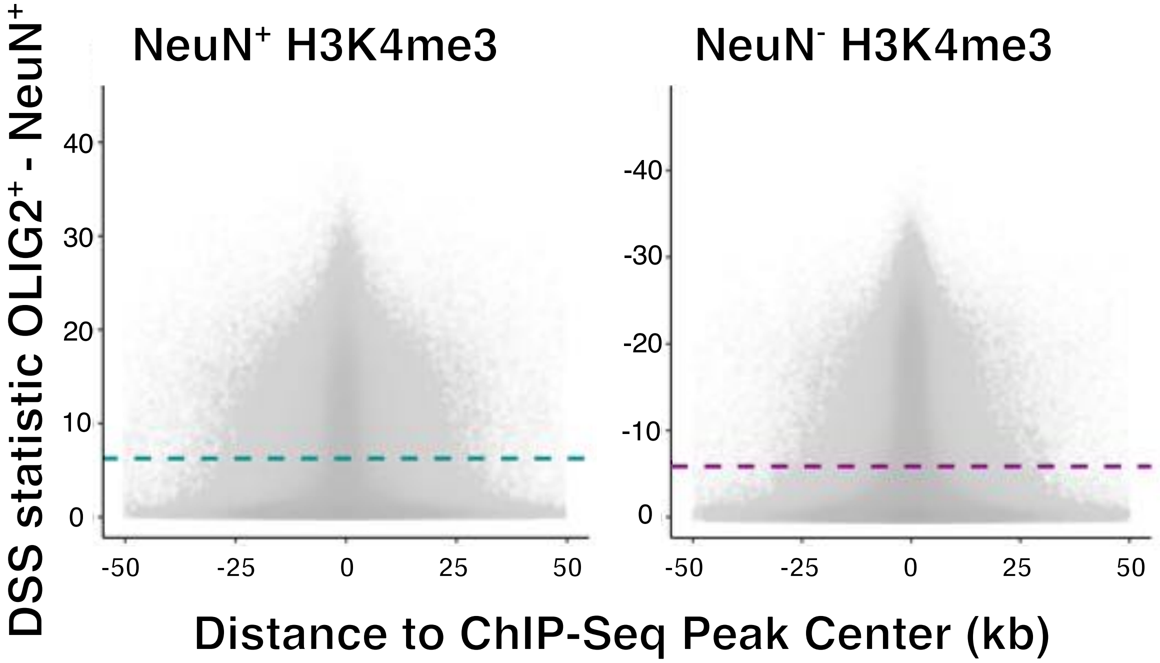

Fig. S3. Non CpG (CH) methylation patterns in brain cell-types. A. Distribution of mean CH methylation levels at gene bodies in NeuN$^+$ (green) and OLIG2$^+$ (magenta) nuclei. **B-C.** Nucleotide context of non CpG (CH) methylation patterns in NeuN$^+$.B. Percentage of sites with methylation info in each context. **C.** Percentage of sites with >1% fractional methylation in each context. **D.** Negative relationship between non-CpG (CH) methylation at gene bodies with gene expression in NeuN$^+$ (Spearman rho = -0.16, P = 6.9e-48).

A

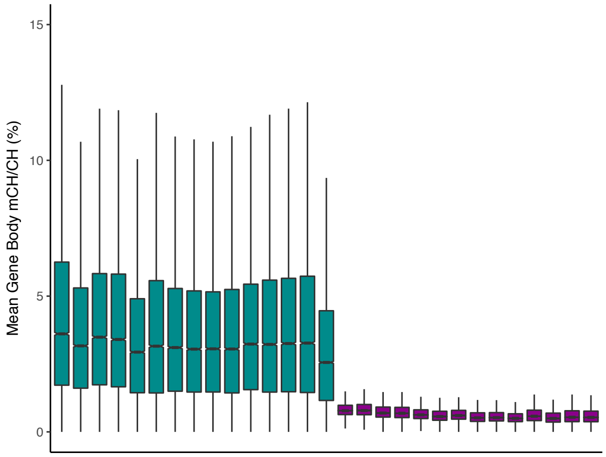

B

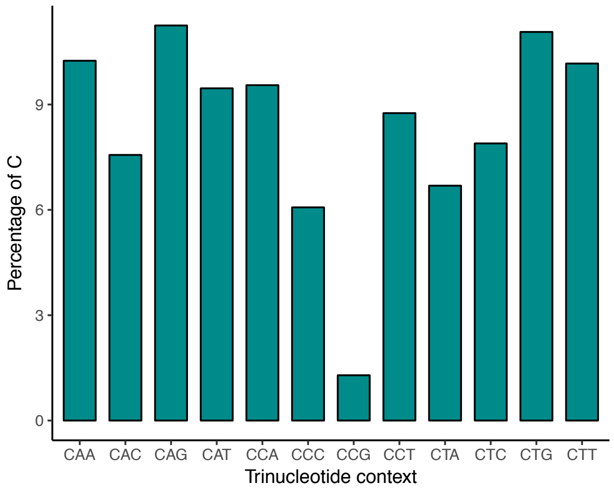

C

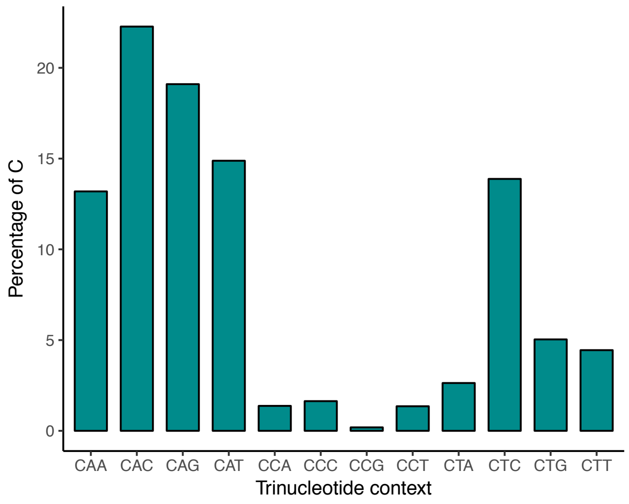

D

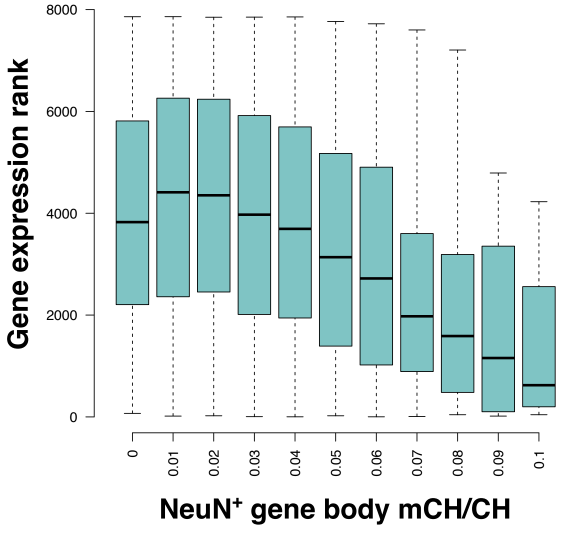

Fig. S4. Concordance between RNA-seq of the current study and adult post-mortem bulk RNA-seq cohort. Showed principal component analysis of gene expression from current study (NeuN/OLIG2), GTEX adult cortical region, GTEX cerebellum and GTEX Whole Blood. Clustering shows concordance of nuclear RNA-seq with cortical regions from adult bulk RNA-seq from post-mortem brain.

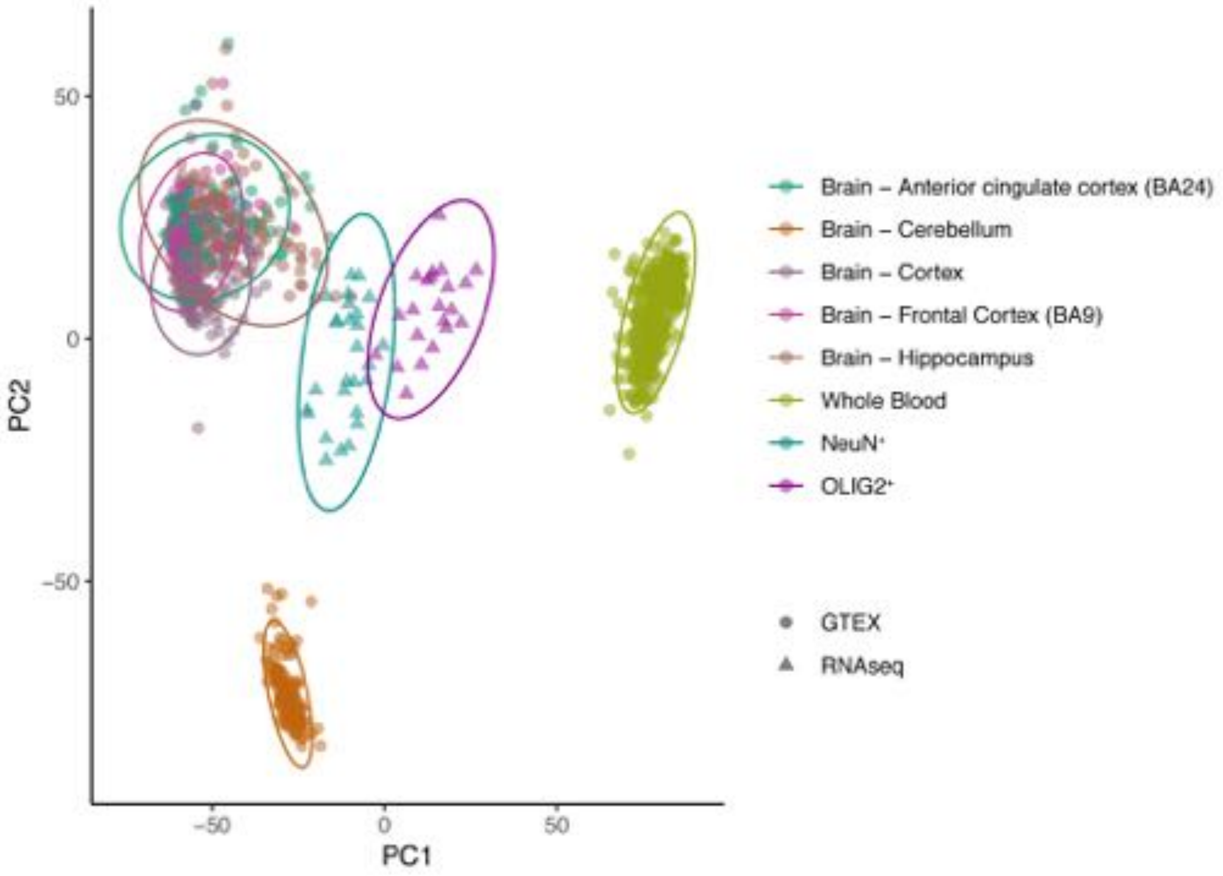

Fig. S5. Cell deconvolution analyses. A, CMC **B**, BrainSeq. Deconvolution is based on Allen MTG data. The y-axis shows the normalized cell proportion. The x-axis shows the cell type detected based on the annotation reported. Both CMC and BrainSeq data are primarily composed of excitatory neurons.

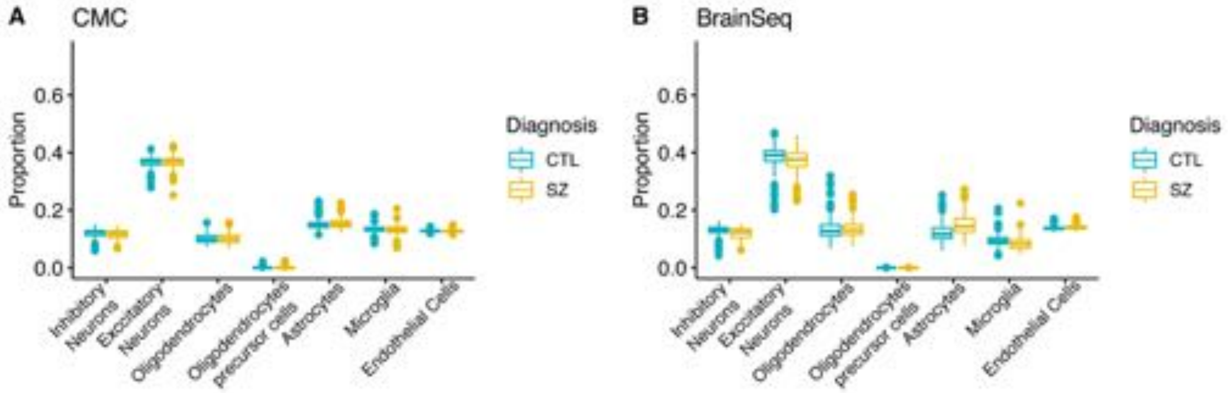

Fig. S6. Correlation values between differential (OLIG2$^+$ vs NeuN$^+$) gene expression and methylation for different genic DMR annotations. All correlations are highly significant (all $P < 2.2 \times 10^{-16}$ except downstream $P = 0.0021$).

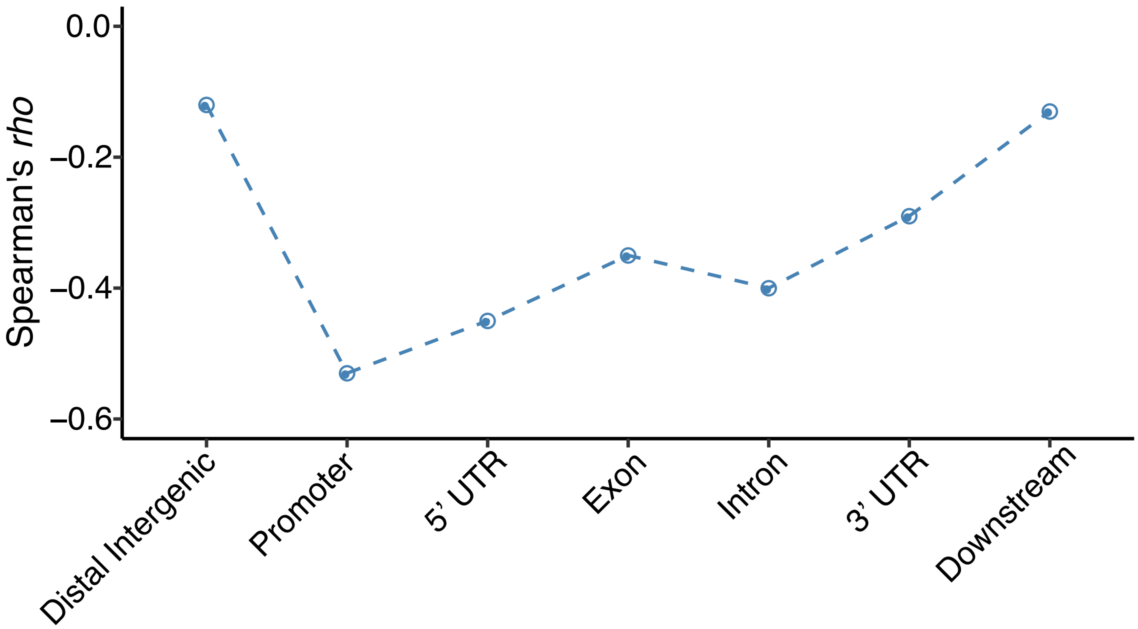

Fig. S7. Power analyses. A. Power calculation heatmap. The X axis show the simulated parametric effect size and the Y-axis shows the observed P-values obtained from case/control linear model. Power refers to the number of times CpGs with a given beta show p-values below the indicated threshold (shown in color legend). **B**. The exploration of the two parameters of a beta distribution to model the distribution of absolute effect sizes (case/control differences). For each of the explored beta distribution (few examples shown in the histograms), we recorded the FDR at top 1,000 DMPs assuming there are 5,000 causal loci for schizophrenia. **C**. The histogram of median effect sizes of causal DMPs found within top 1,000 p-values in the simulations at FDR 50%. The average of median values is around 5.8% (indicated with a vertical line).

A.

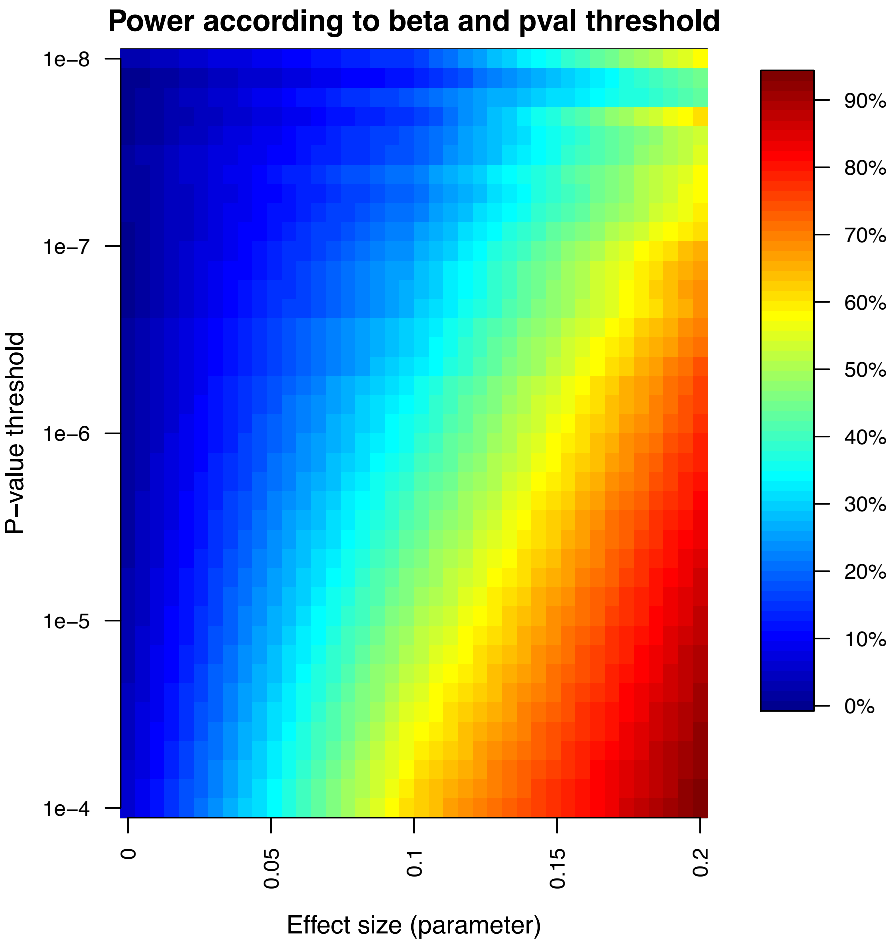

B.

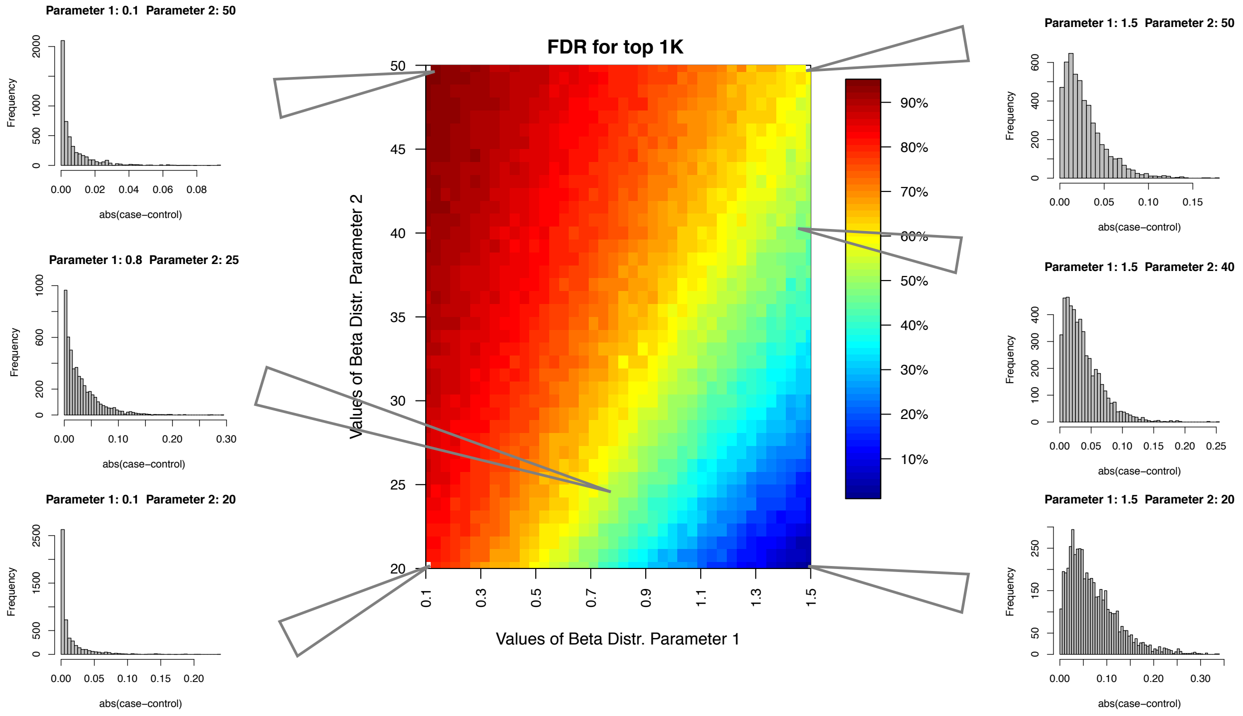
Parameter 1: 0.1 Parameter 2: 50
Frequency
abs(case−control)
Parameter 1: 0.8 Parameter 2: 25
Frequency
abs(case−control)
Parameter 1: 0.1 Parameter 2: 20
Frequency
abs(case−control)
FDR for top 1K
Values of Beta Distr. Parameter 2
Values of Beta Distr. Parameter 1
90%
80%
70%
60%
50%
40%
30%
20%
10%
Parameter 1: 1.5 Parameter 2: 50
Frequency
abs(case−control)
Parameter 1: 1.5 Parameter 2: 40
Frequency
abs(case−control)
Parameter 1: 1.5 Parameter 2: 20
Frequency
abs(case−control)

C.

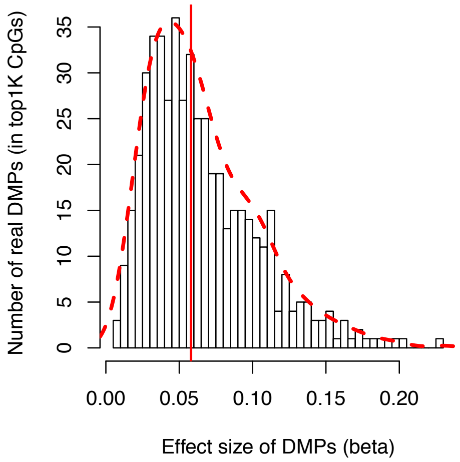
Number of real DMPs (in top1K CpGs)
Effect size of DMPs (beta)

Fig. S8. Permutation analyses. A-B. Absolute methylation difference between control and schizophrenia in DSS significant hits (at FDR < 0.2) compared to hits obtained at FDR < 0.2 in 100 case-control permuted datasets in NeuN+ (A) and OLIG2+ (B). Methylation differences. **C.** Distribution of hypo and hypermethylated loci associated with schizophrenia (szDMPs) at FDR < 0.2, and those showing top 1,000 *P* values. **D-E.** Observed (dashed lines) ratios of hypermethylated hits in schizophrenia versus control at top 1,000 szDMPs in each cell-type compared to the datasets generated by permutation of case/control labels 100 times per cell-type.

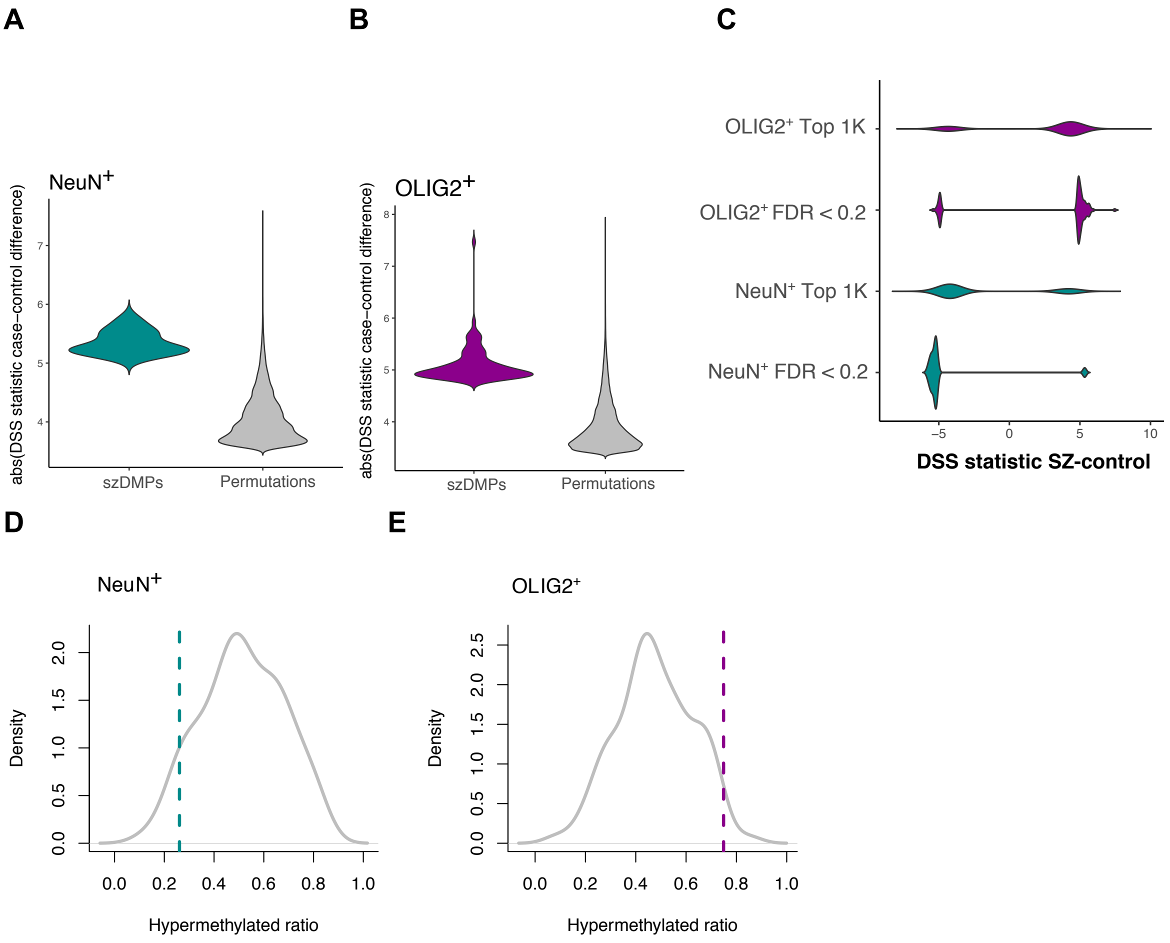

Fig. S9. Correspondence between fractional methylation values in WGBS and targeted high coverage bisulfite sequencing experiments in the same subset of individuals. **A.** Targeted loci (16 CpGs). **B.** Targeted and adjacent loci (66 CpGs). Each dot represents an individual and a locus.

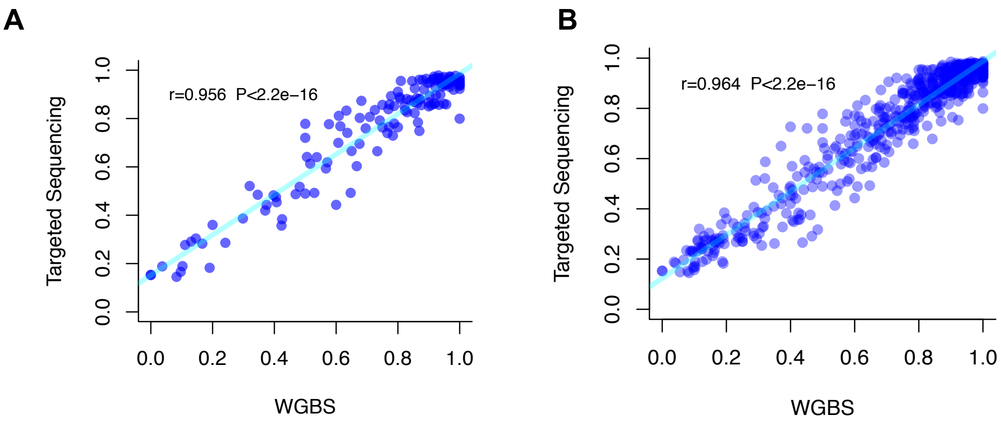

Fig. S10. Case control differences in validation experiments. **A**. Targeted loci (szDMPs) in the validation cohort. 14 out of 16 loci (87.5%) show consistent sign between WGBS and targeted experiments. **B**. Targeted loci in the subset of samples that are new in the validation cohort (biological replicates). 10 out of 16 loci (62.5%) show consistent sign between WGBS and targeted experiments. **C.** Targeted and adjacent loci in the region in the validation cohort. 54 out of 66 loci (81.8%) show consistent sign between WGBS and targeted experiments. **D**. Targeted and adjacent loci in the region in the subset of samples that are new in the validation cohort (biological replicates). 45 out of 66 loci (68.18%) show consistent sign between WGBS and targeted experiments.

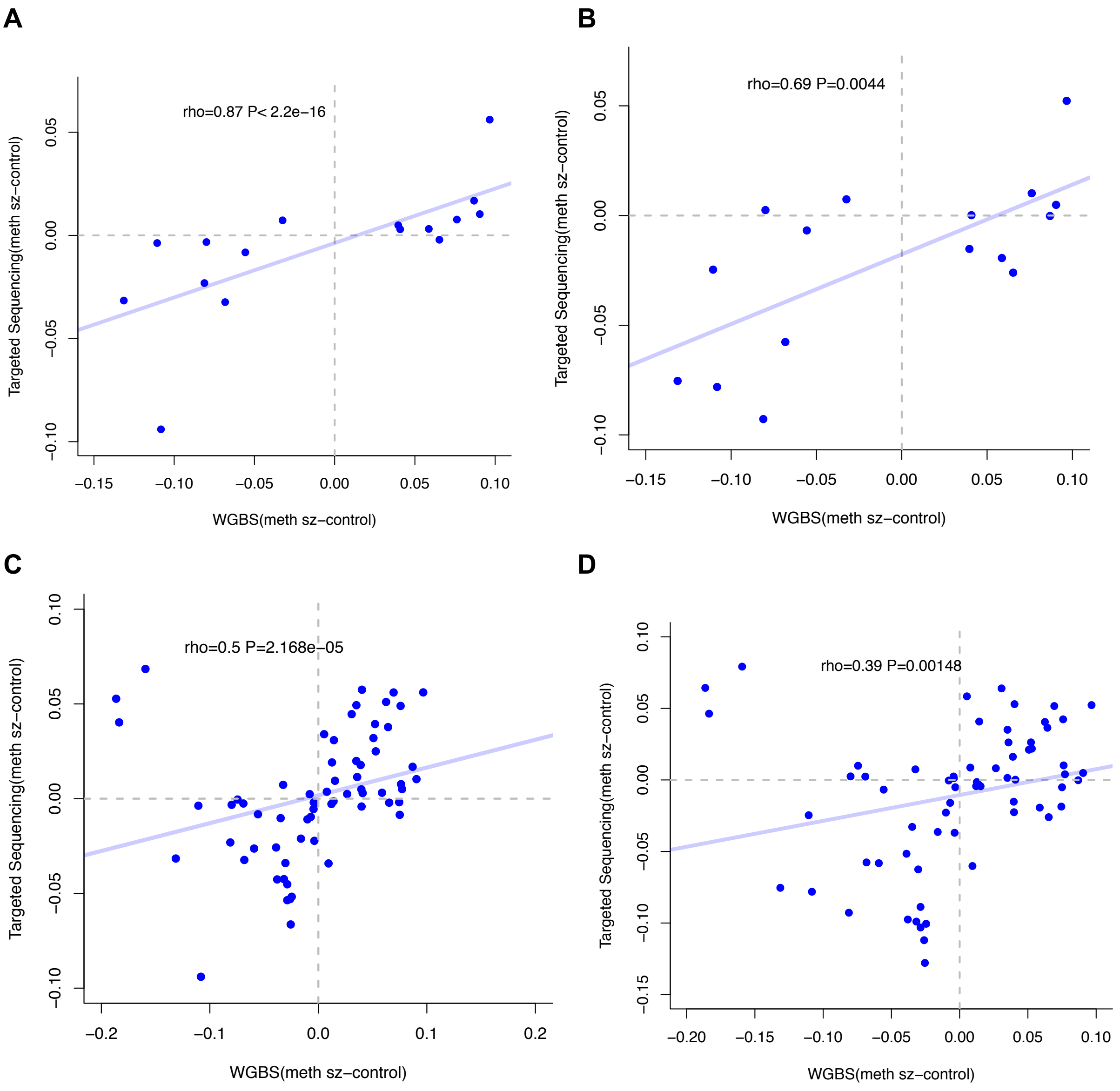

Fig. S11. Cell-type differences at szDMPs. The Y-axis depicts absolute cell-type ($OLIG2^+$ vs $NeuN^+$) differences from the DSS statistics. The first set (red) includes sites that are identified as significantly differentially methylated between cell types (cell-type DMPs), which accordingly show large difference. The second column (gray) depicts the rest of the genome (no-DMPs). The third column (blue) shows that sites that tend to be differentially methylated between schizophrenia and control (top 1,000 sites from each cell-type according to DSS *P* values) exhibit large cell-type differences. In particular, these sites show significantly greater differences compared to those reported as significantly differentially methylated between schizophrenia and control from Illumina450K methylation arrays by Jaffe et al. (PMID 26619358) (shown in the fourth column).

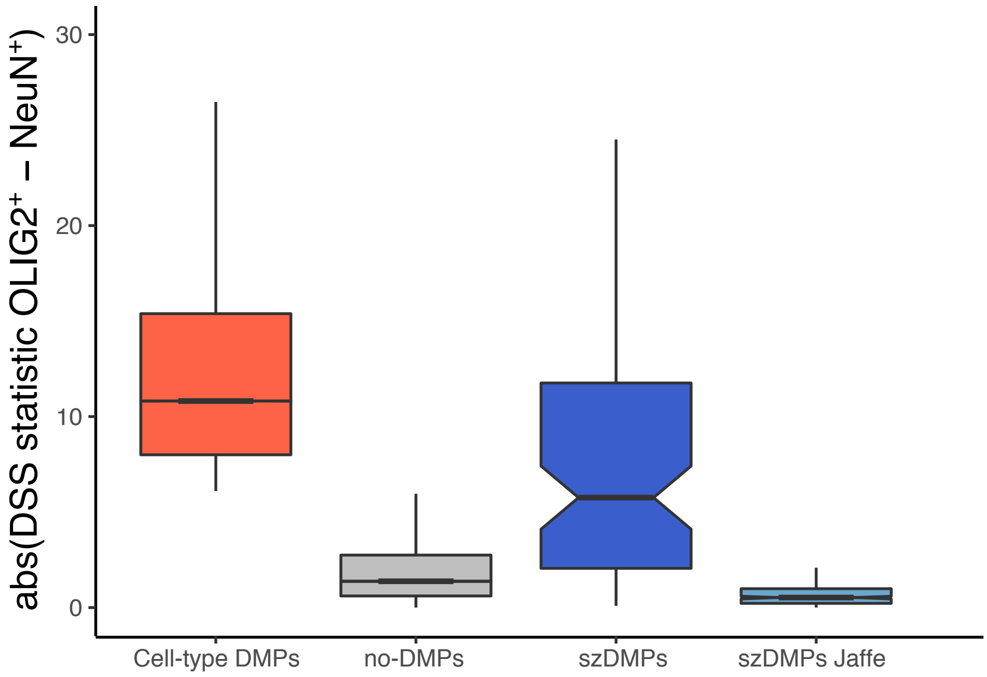

Fig. S12. Functional enrichment of top 1,000 szDMPs. **a.** Functional category enrichment of genes harboring top 1,000 szDMPs based on ToppGene. GO: Biological Process (cyan), GO: Molecular Function (gray), GO: Cellular Component (yellow) and Disease (red) categories shown. **b**. Number of top 1,000 szDMPs co-localizing with transcription factor ChIP-Seq data from ENCODE (vertical colored lines) compared to 100 sets of non-szDMPs (gray distributions). **c.** Frequency of specific transcription factors in top 1,000 szDMPs (colored dots, NeuN^{+} in green and OLIG2^{+} in magenta) and in 100 sets of non-szDMPs (gray dots). We calculated enrichments as the ratio between observed frequency and the mean frequency of control sets. Only TFs with significant enrichments are shown (HDAC8=4.4; NR2F2=2.07; ZBTB7A=2.6; ZEB1=3.8_ SMARCA4 =3.7; all P < 0.05).

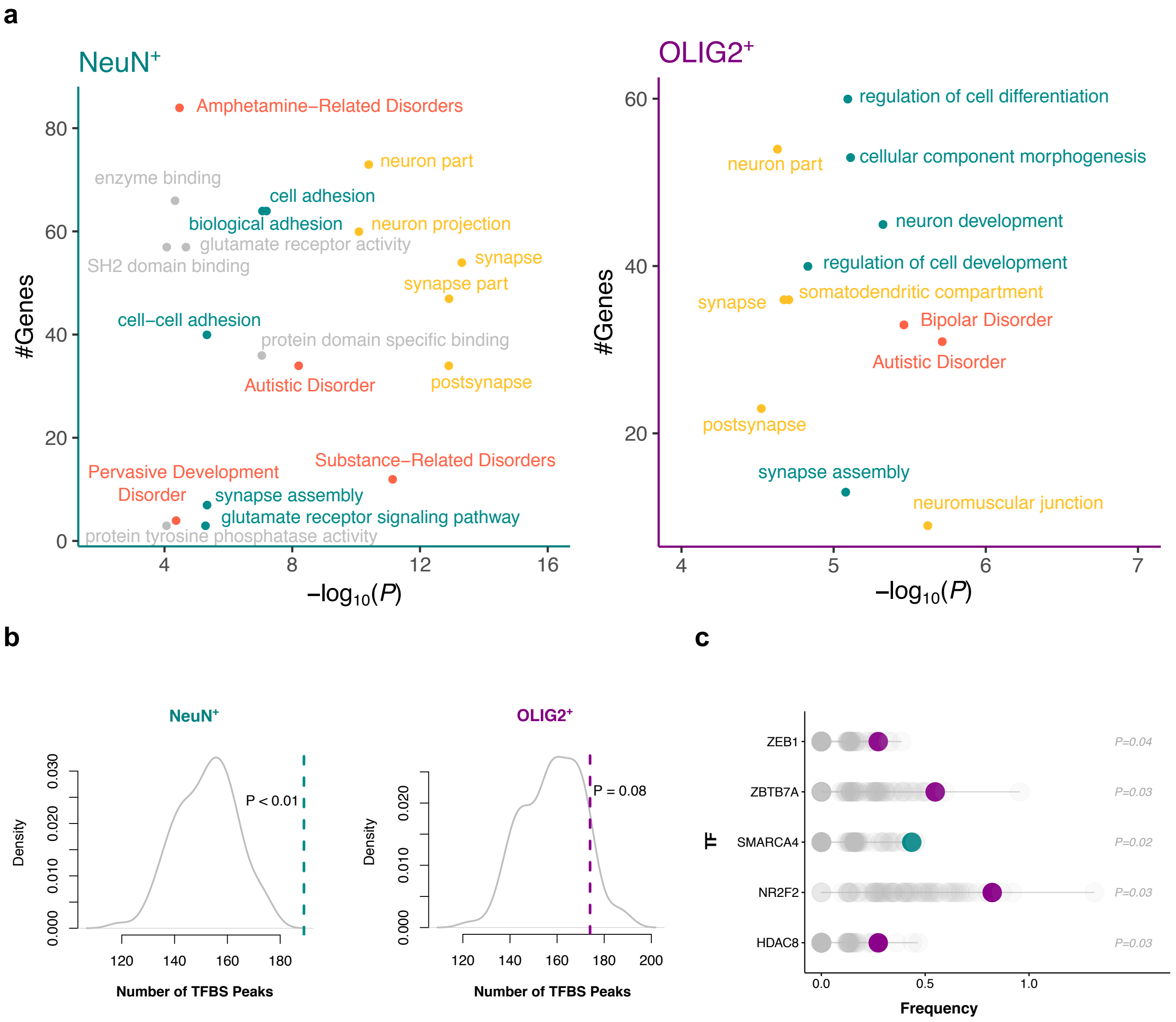

Fig. S13. Overview of workflow for WGBS and WGS data processing and differential methylation analyses.

	Step	Step Description
1	**WGBS library preparation**	DNA extraction<br>Bisulfite conversion of DNA<br>Library preparation and QC
2	**Sequencing**<br>(Hi-SeqX , HiSeq2500)	Paired-end Illumina sequencing
3	**Adapter and quality trimming**<br>(Trim Galore v.0.4.1, FastQC)	Trim low quality base-pairs at read ends<br>Trim adapters sequenced within reads<br>Evaluate read quality
4	**PhiX removal**<br>(Bowtie2 v2.2.7)	Removal of phiX spike-in control
5	**Mapping**<br>(Bowtie v1.1.2, Bismark v0.14.5)	Map reads to human hg19 genome
6	**Deduplication**<br>(Bismark v0.14.5)	Remove potential PCR duplicates
7	**Methylation extraction**<br>(Bismark v0.14.5)	Estimate per base fractional methylation
8	**Differential methylation analyses**	

	Step	Step Description
1	**WGS library preparation**	DNA extraction<br>Library preparation and QC
2	**Sequencing**<br>(Hi-SeqX , HiSeq2500)	Paired-end Illumina sequencing
3	**Adapter and quality trimming**<br>(Trim Galore v.0.4.1, FastQC)	Trim low quality base-pairs at read ends<br>Trim adapters sequenced within reads<br>Evaluate read quality
4	**Mapping**<br>(bwa v 0.7.4-r385)	Map reads to human hg19 genome
5	**Deduplication**<br>(Picard v.1.93)	Remove potential PCR duplicates
6	**Variant Discovery**<br>(GATK v3.5)	SNP calling

Exclusion of polymorphic CpG sites (C->T and G->A)

Fig. S14. Genetic ancestry of studied individuals. A. Principal component plot of the 54 individuals included in this study. **B.** Percentage of variance explained by the PCA in panel A. **C**. Principal component plot of the individuals included in this study and additional 210 individuals from 4 reference populations from HapMap Project (60 CEU, 90 CBH/JPT and 60 YRI). After LD pruning (r^2>0.2) with SNPRelate R package (Zheng, et al. 2012), we used 66,667 autosomal polymorphic SNPs on this analysis. **D.** Percentage of variance explained by the PCA in panel C.

A

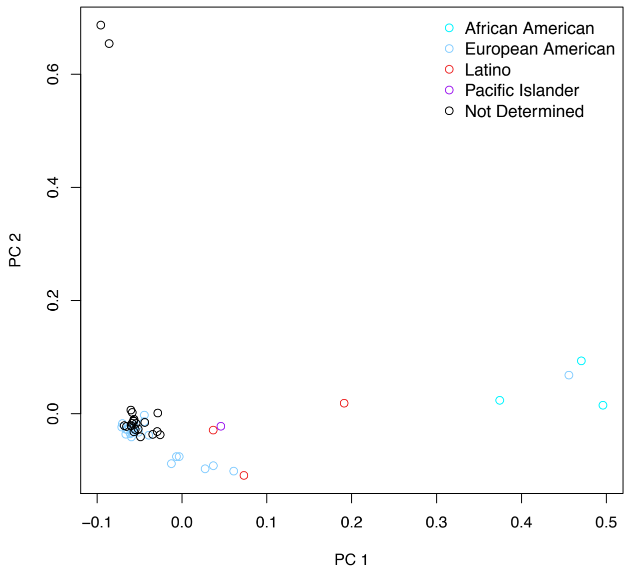

B

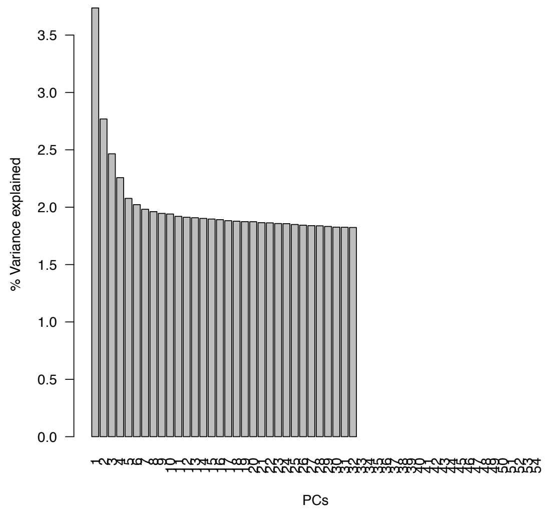

C

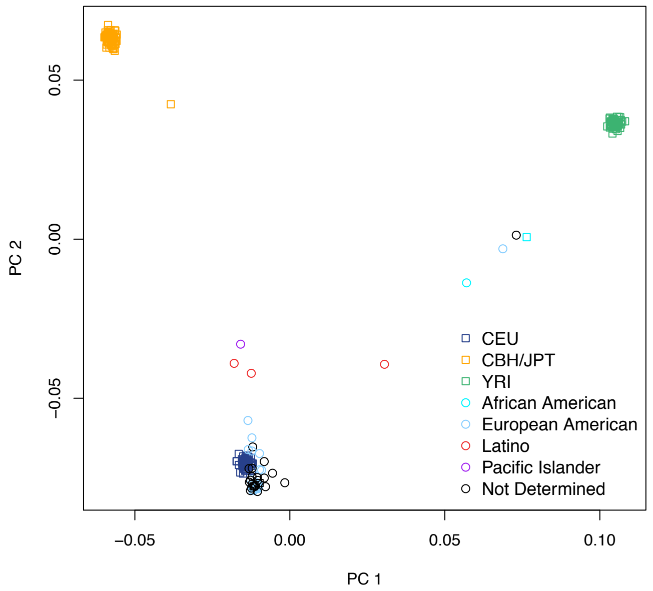

D

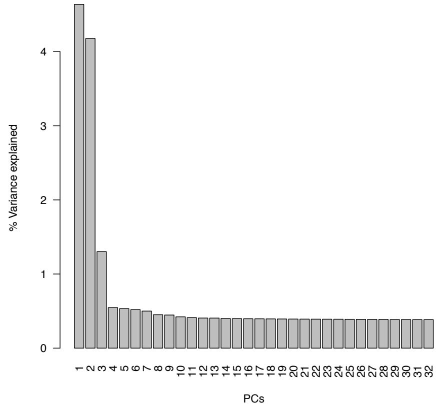

Fig. S15. Distribution of covariates with disease status in NeuN$^+$ and OLIG2$^+$ WGBS samples (controls: dark gray, schizophrenia: light gray). **A**. Brain bank **B**. Post-mortem interval class **C**. Hemisphere **D**. Age class **D**. Sex **F**. Conversion rates.

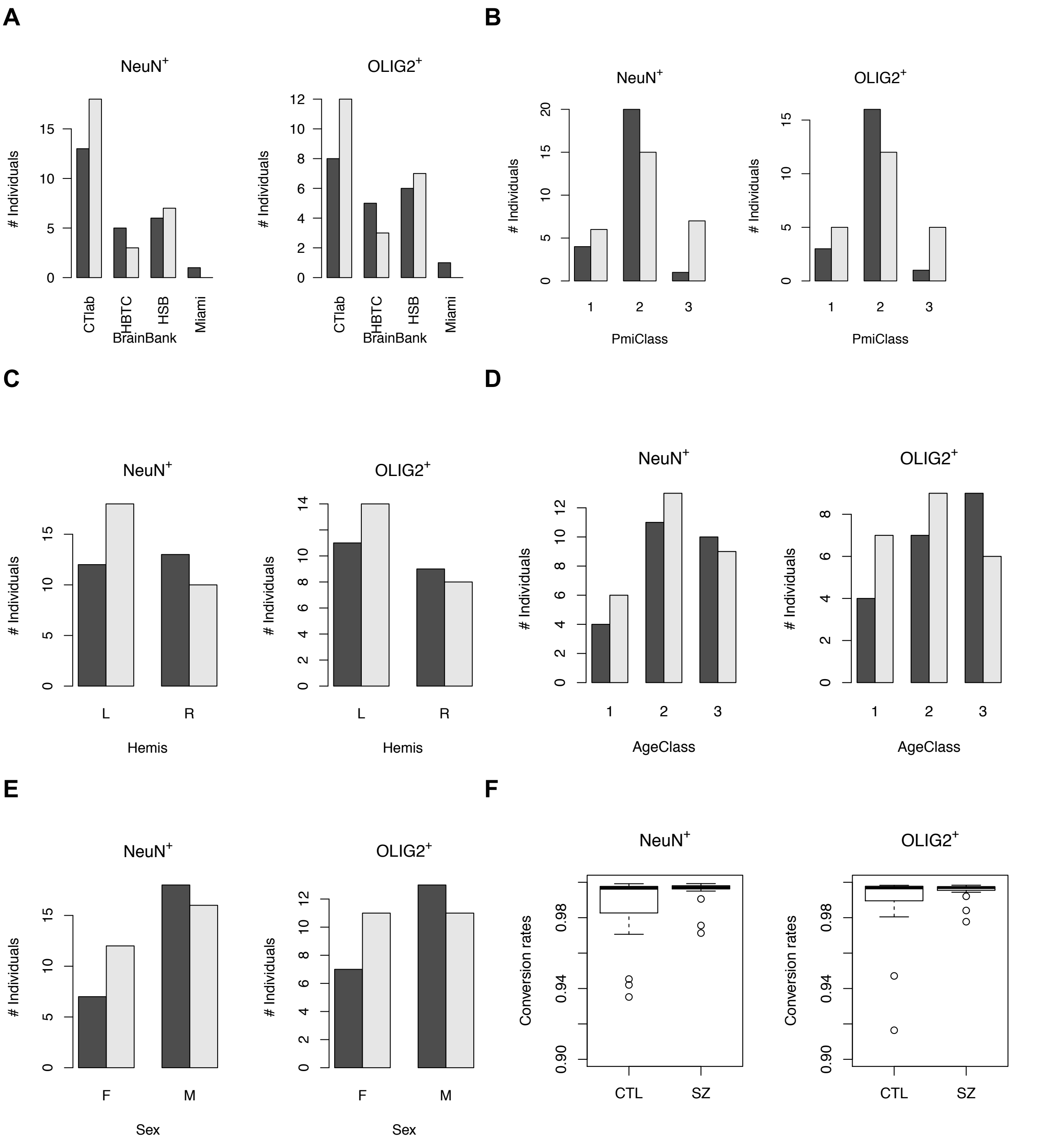

Fig. S16. Effect of coverage at szDMP discovery. A. Quantile-quantile plots of genome-wide schizophrenia vs control *P* values for DSS (blue) and linear model (gray) using ~25 million CpG sites. For plotting purposing only *P* < 0.01 are shown. **B.** Quantile-quantile plots of *P* values for NeuN^{+} at sites with low (<5x, light empty dots) and high coverage (>20x, dark filled dots) in DSS (left) and linear model (right). DSS *P* values show systematic depletion at sites with low coverage compared to the linear model, which does not specifically model read coverage.

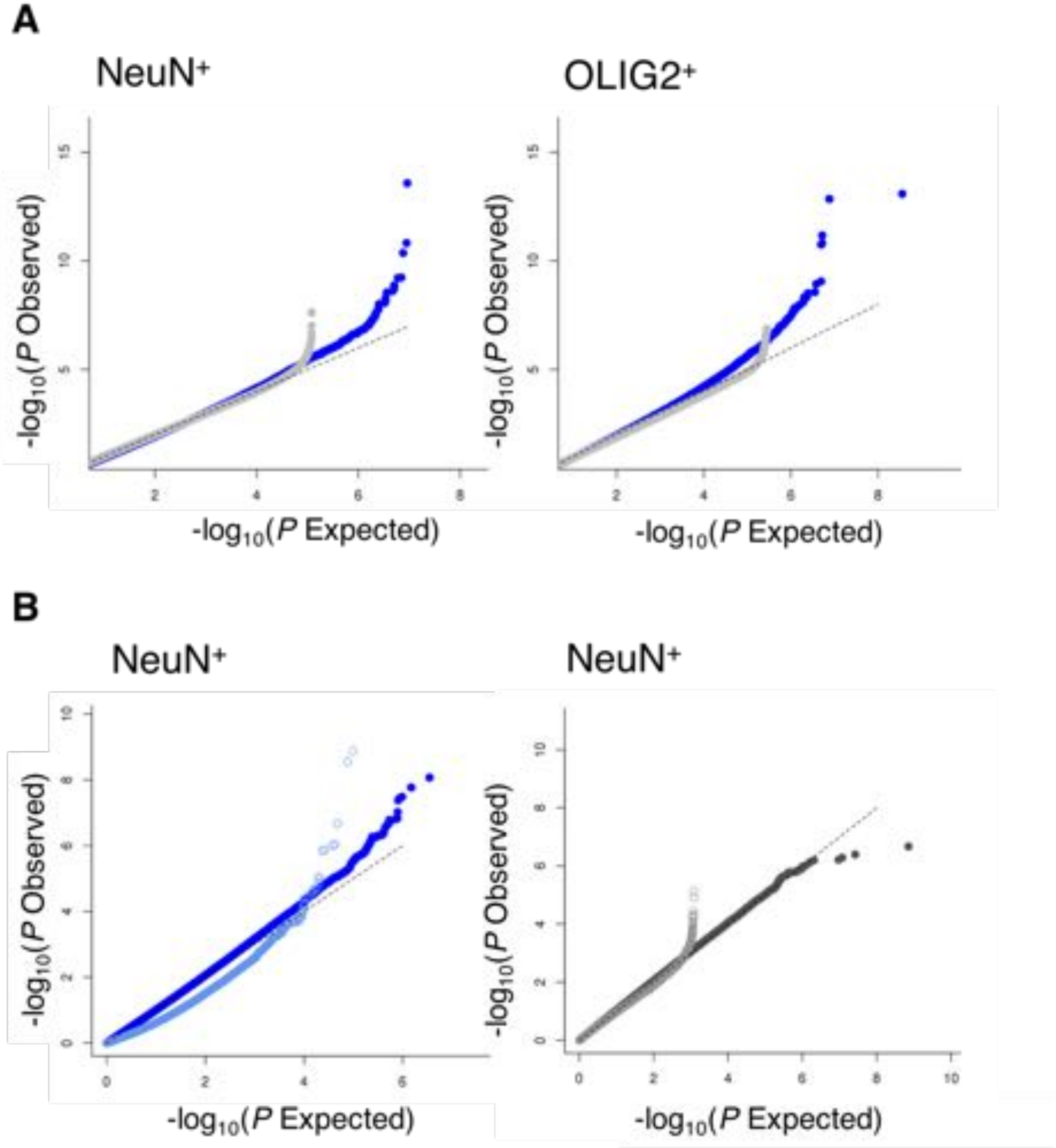

Fig. S17. QQ-plots of DSS sz-association *P* values at random loci (top row) vs high-hmC sites in adult brain (bottom row).

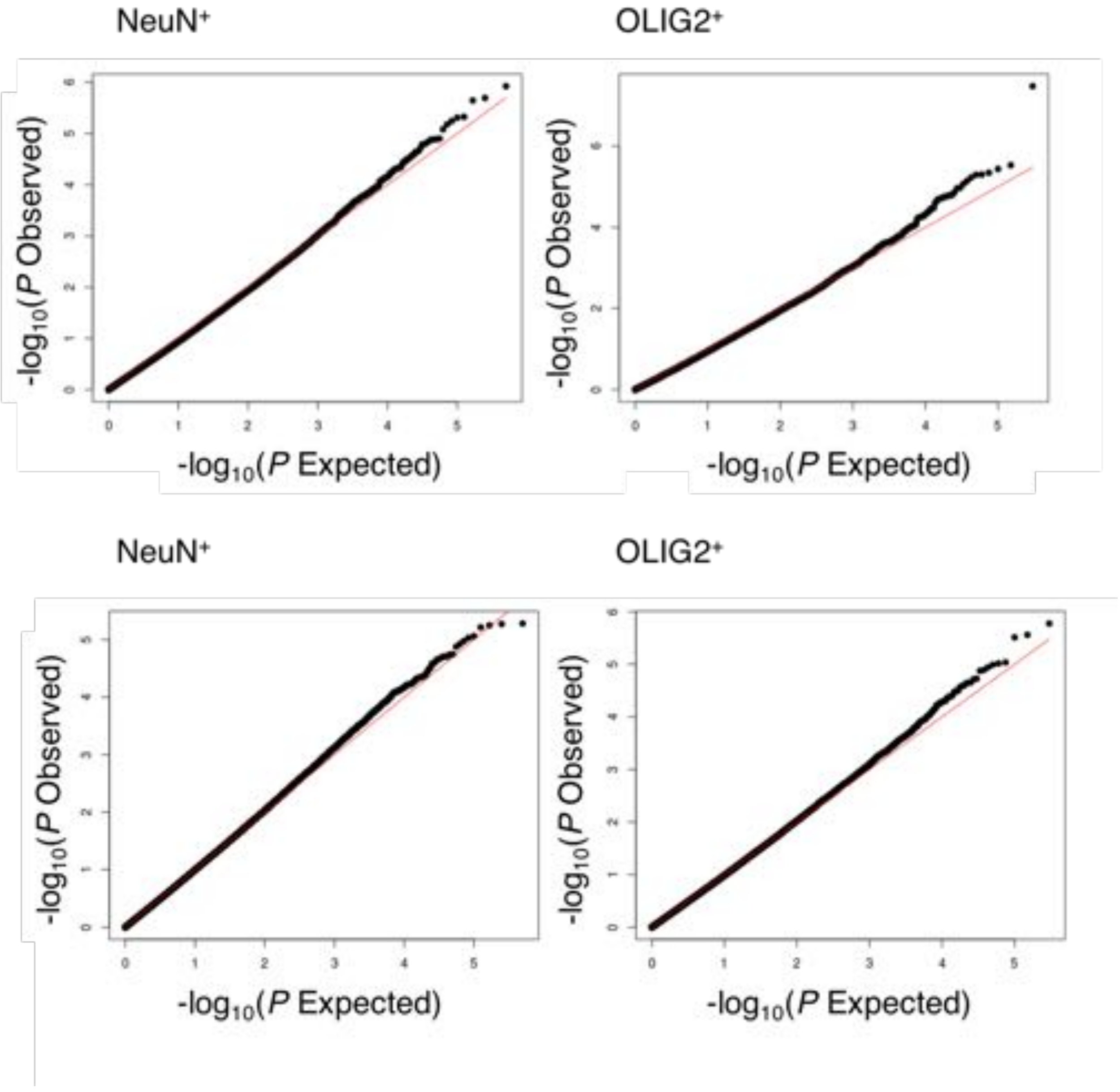

Fig. S18. Major QC metrics of the presented study. Each panel corresponds to major statistics from picard tool (http://broadinstitute.github.io/picard/).Distribution is represented for NeuN+ and OLIG2+ divided by Control and Schizophrenia samples.

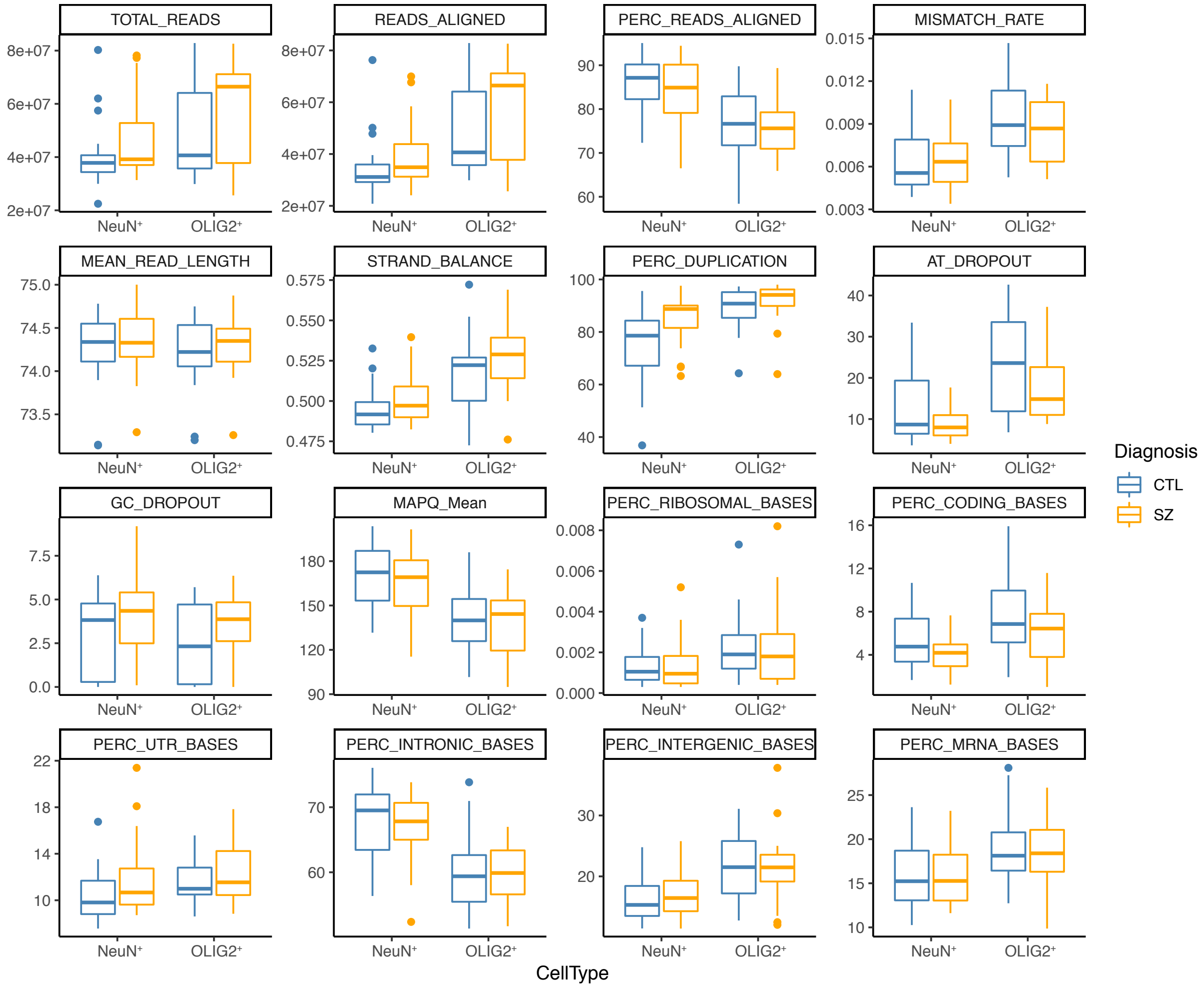

Fig. S19. Additional QC metrics of the presented study. A) gene body distribution, **B)** read distribution after filtering steps, **C)** read distribution per features normalized by length (kb).

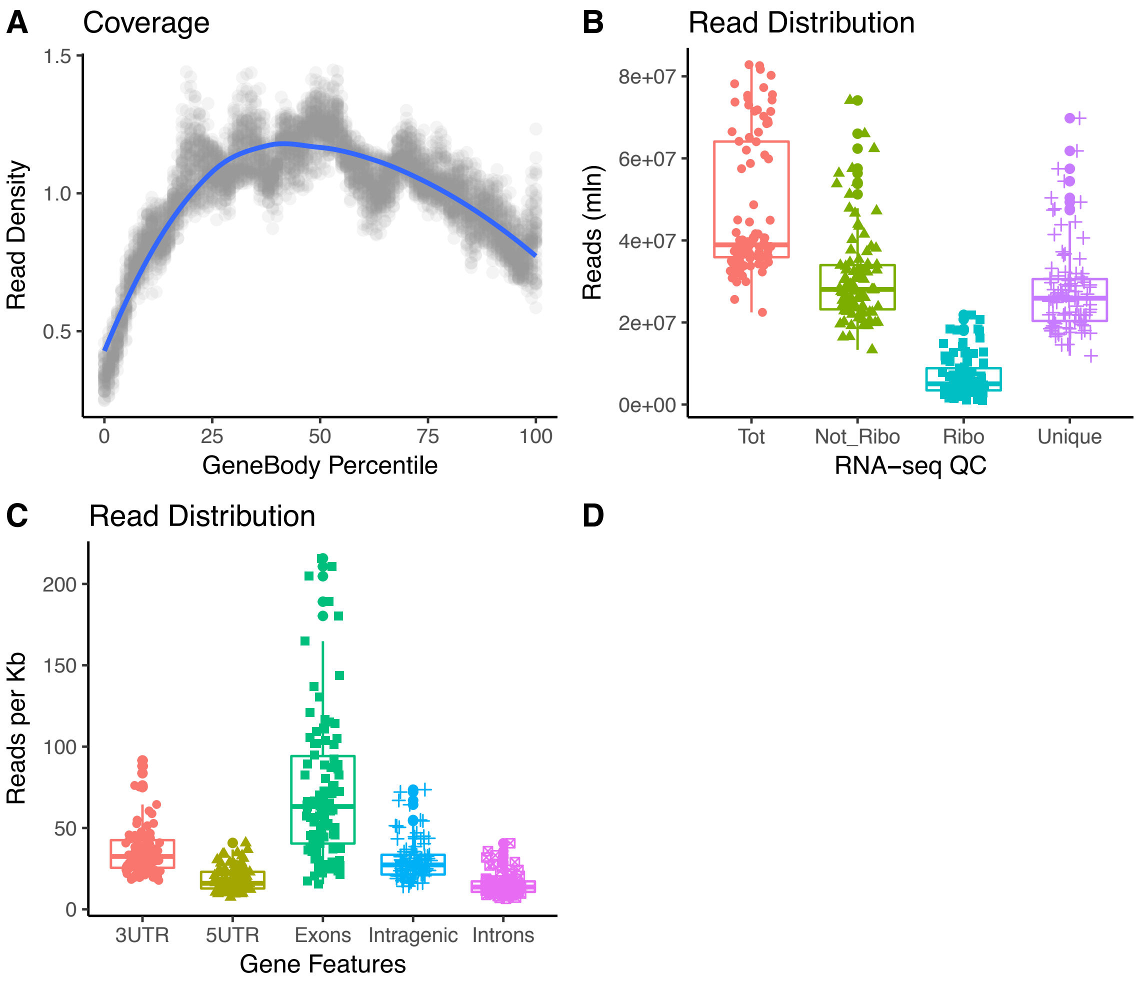

Fig. S20. Quality Control for the variance explained by the confounder. A linear model was applied to determine the effect of potential confounder on the subject studies. *P* values are defined by ANOVA test.

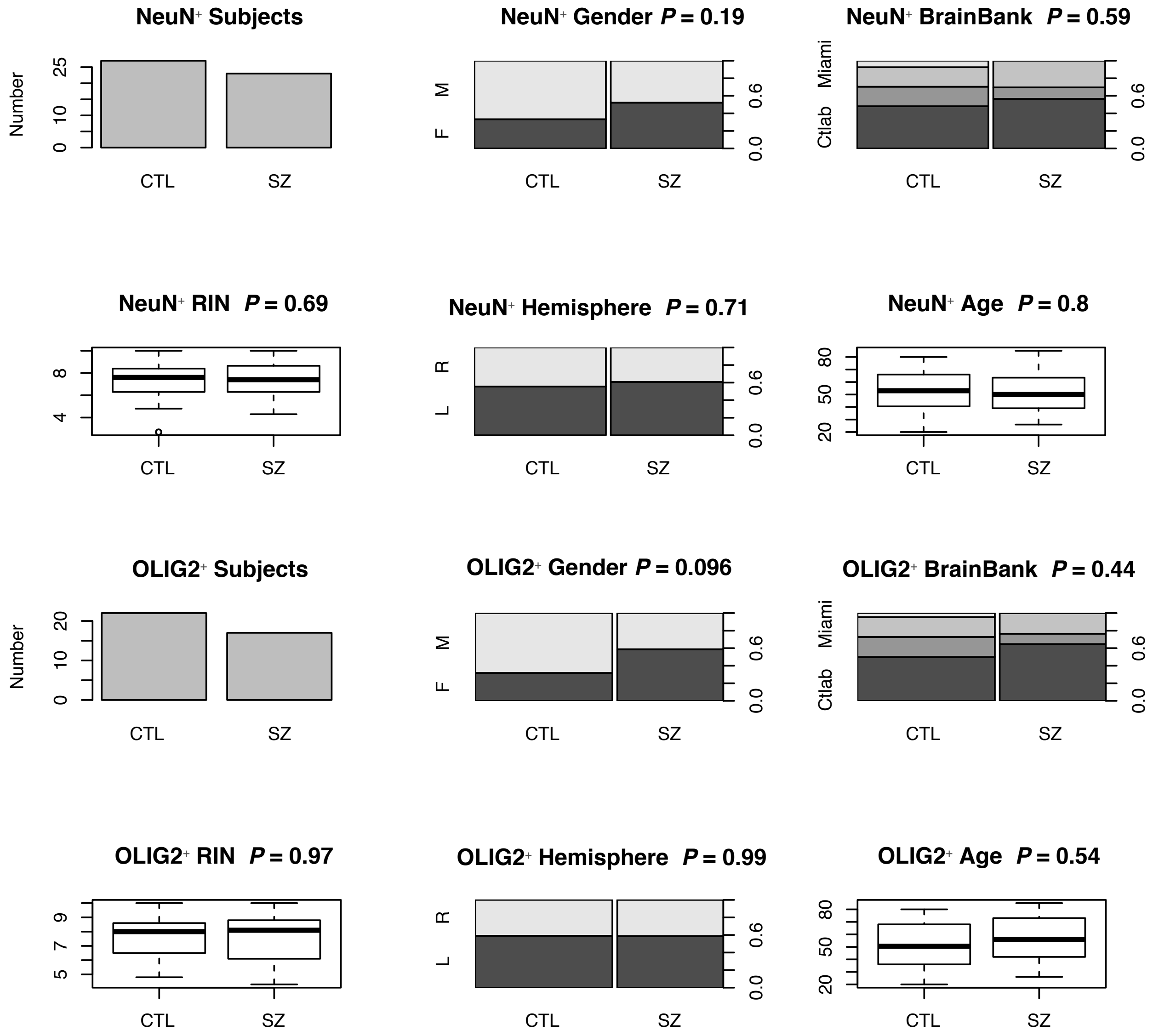

Supplement: Supplementary file 3 — Supplementary Figures S1–S20. (PDF 1643 kb) [file 13059_2019_1747_MOESM3_ESM.pdf]
